# In-silico characterization of GABAT protein found in gut-brain axis associated bacteria of healthy individuals and multiple sclerosis patients

**DOI:** 10.1016/j.sjbs.2024.103939

**Published:** 2024-02-02

**Authors:** Nadia Hussain, Fatima Muccee

**Affiliations:** a Department of Pharmaceutical Sciences, College of Pharmacy, Al Ain University, Al Ain Campus, Al Ain 64141, United Arab Emirates; b AAU Health and Biomedical Research Center, Al Ain University, Abu Dhabi Campus, Abu Dhabi P. O. Box 112612, United Arab Emirates; c School of Biochemistry and Biotechnology, University of Punjab, Lahore 52254, Pakistan

**Keywords:** Multiple sclerosis, Uniprot, *Faecalibacterium*, Dysbiosis, 4-aminobutyrate transaminase, Gut-brain axis, Postbiotics

## Abstract

**Background:**

Multiple sclerosis (MS) is a neurodegenerative disease characterized by inflammation and demyelination of neurons. There is evidence to suggest that level of a neurotransmitter gamma-aminobutyric acid (GABA), due to the degradation by γ-aminobutyric acid transaminase (GABAT), is reduced in certain areas of the brain in MS patients. MS is always accompanied by gut bacteria dysbiosis. In healthy individuals, *Faecalibacterium* sp. while in MS patients *A. calcoaceticus, Clostridium* sp. and *S. typhimurium* are found abundantly. Although all these microbes produce GABAT but only in MS patients this enzyme significantly degrades GABA.

**Objective:**

Present study is an attempt to characterize the GABAT protein sequences of these bacteria.

**Methodology:**

Sequences of GABAT protein were retrieved from Uniprot database. Sequences were analyzed by Protparam, Gneg-mPLoc, SOSUI, PFP-FunDSeqE, Pepwheel program, PROTEUS and Alphafold and SAVES servers, MEME suite and HDOCK server.

**Results:**

In healthy individuals gastrointestinal tract (GIT) bacteria, GABAT protein was present in inner-membrane with α helix content (61 and 62%) and β sheet content (5%), 4-helical cytokines functional domains. It has greater number of B-cell epitopes and more complex 3D configuration as compared to MS patients GIT bacterial enzymes.

**Conclusion:**

Present study might enable us to modify the GABAT encoding gene and enzyme through site-directed mutagenesis in pathogenic bacteria thus reducing their potential of causing MS.

## 1 Introduction

Gut-brain axis (GBA) is a bidirectional communication system between the central nervous system (CNS) and the gut microbiota which consists of four components i.e. gut inhabiting bacteria, intestinal barrier associated with the entry of microbial metabolites into organism, enteric nervous system (ENS) and vagus nerve (Parodi and Kerlero de Rosbo, 2021, Thirion et al., 2023). This connection is maintained through postbiotics or bacterial metabolic products e.g. short chain fatty acids (SCFA) including acetate, butyrate and propionate, gamma-aminobutyric acid (GABA), dopamine, melatonin, tyramine (Tyr), histamine (His), β-phenylethylamine (Phe), melatonin (Trp), serotonin / triptamin (Trp), tryptophan metabolites, saturated and unsaturated long chain fatty acids (LCFA), bile acids and bacterial components like lipopolysaccharides (LPS) (Lavelle and Sokol, 2020, Mazzoli and Pessione, 2016). Major purpose of this axis is the homeostasis of CNS, gastrointestinal tract (GIT) and the microbes. Brain-gut interaction occurs via multiple pathways i.e. type 1 interferon (IFN-1), nuclear factor kappa B (NF-kB) and inflammasome signaling pathway (Ma et al., 2019).

Due to the association of gut bacteria with innate pathways of immune system and various physiological mechanisms of CNS, their dysbiosis might lead to neurodegenerative pathologies (Ma et al., 2019). One of these pathological conditions is multiple sclerosis (MS) (Cheng et al., 2019). MS is a chronic autoimmune and neurodegenerative disease characterized by inflammation and demyelination of neurons particularly in the CNS (Farshbafnadi et al., 2021). There is evidence to suggest that GABA level is reduced in several areas of the brain in individuals with MS. GABA is a neurotransmitter that plays an important role in regulating neuronal excitability in the CNS. Demyelination of neurons results in memory impairment, sensory and motor deficits such as muscular spasms or stiffness and numbness in legs. It also leads to cognitive impairment including difficulty with memory, attention, and problem-solving. MS is a consequence of interplay between environment and organism’s genetic makeup (Schapiro, 2014). The MS causing factors include more than 200 mutations, sanitation, antibiotics use, lifestyle, vitamin D deficiency, Epstein-Barr virus and childhood and adulthood obesity (Belbasis et al., 2020). Additionally, disturbance in gut inhabiting bacteria, postbiotics, permeability of intestine and nervous system activities have also been observed as contributing factors of MS (Buscarinu et al., 2017, Camara-Lemarroy et al., 2020, Escribano et al., 2017, Ghezzi et al., 2021, Kriesel et al., 2019, Laman et al., 2020, Mirza and Mao-Draayer, 2017).

Studies have reported changes in the populations of various bacteria in the GIT of MS infected individuals. These changes include both increases and decreases in bacterial populations compared to healthy individuals. Bacteria that show a reduction in individuals with MS include *Faecalibacterium* sp., *Bacteroidetes*, *Akkermansia muciniphila*, *Bifidobacterium*, and *Clostridia* clusters XIVa and IV. (Chen et al., 2016, Miyake et al., 2015, Mowry et al., 2012, Pellizoni et al., 2021). Conversely bacteria like *Streptococcus*, *Parabacteroides distasonis*, *Acinetobacter calcoaceticus*, *Clostridium* sp. and *Salmonella typhimurium* are reported to increase in MS patients (Cekanaviciute et al., 2017, Zeng et al., 2019, Zhou et al., 2022). One gut inhabiting bacterium *Flavonifractor* sp. is GABA fermenting microbe and hence, have association with neurological disorders (Strandwitz et al., 2019). Four bacteria are addressed in present study. Among these *Faecalibacterium* sp. is reported to decrease in MS patients gut while *Acinetobacter calcoaceticus*, *Clostridium* sp. and *Salmonella typhimurium* are reported to increase in number (Castillo et al., 2016, Cekanaviciute et al., 2016, Chen et al., 2016, Jangi et al., 2015, Liu et al., 2022, Mowry et al., 2012, Noto and Miyake, 2022, Parker et al., 2020, Zhou et al., 2022).

**Fig. 1:**
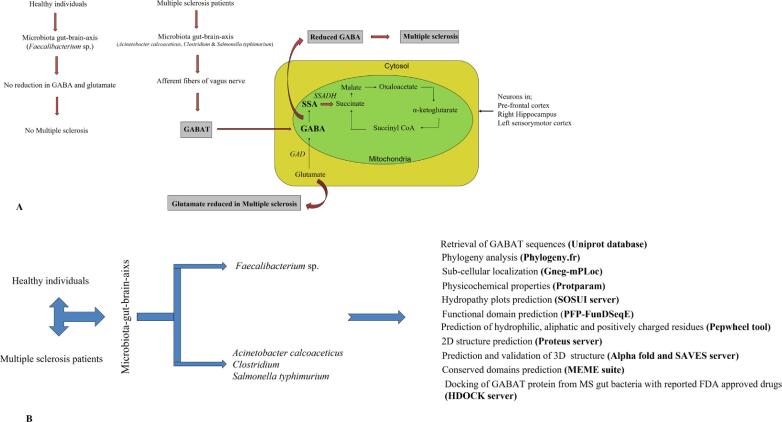
Graphical representation of present study (Ramachandran V. S., 2002). (A)Mechanism of action of GABAT in microbiota-gut-brain axis of healthy individuals and multiple sclerosis patients (B) Layout of methodology involved in present study

GABA is an inhibitory neurotransmitter produced in GABAergic neurons from L-glutamate in the presence of L-glutamic acid decarboxylase (Chang et al., 2003, Petroff, 2002). It is an important neurotransmitter that contributes to processes such as cortical adaptation, synaptic plasticity, and neural reorganization. (Wu and Sun, 2015). The degradation of GABA begins with the action of γ-aminobutyric acid transaminase (GABAT), which converts it into succinic semialdehyde. This is then further metabolized into succinate within the mitochondria, which enters the Krebs cycle. Apart from its role as a neurotransmitter, GABA also functions as an anti-inflammatory molecule. Under normal circumstances, the amount of GABA being degraded is replenished through the conversion of L-glutamate. (Ramachandran V. S., 2002). Studies have proved that gut dwelling bacteria play a significant role in MS. Mechanisms through which these bacteria contribute to MS may include, weakening of immune system and induction of autoimmune myelin sheath deterioration, production of GABA degrading enzyme GABAT, GABA fermentation and initiating inflammation by inhibiting anti-inflammatory IL10 expressing human CD4^+^CD25^+^T cells (Cekanaviciute et al., 2017, Ochoa-Repáraz et al., 2018, Shahi et al., 2017, Strandwitz et al., 2019). A high abundance of gut bacteria producing GABAT) could result in a reduced amount of GABA in the neurons of the prefrontal cortex, left sensorymotor cortex, and right hippocampus, potentially contributing to the development of MS. In such instances, there may also be a decrease in the levels of glutamate, which is another important neurotransmitter in the nervous system (Nantes et al., 2017). Degradation of GABA by GABAT cannot be fully compensated, which can result in a significant reduction in GABA levels (Fig. 1A). One of the major consequences of this GABA reduction is physical disability in MS patients. (Cawley et al., 2015).

Taking in account the involvement of GABAT enzyme in the reduction of GABA levels as well as the abundance of *Acinetobacter calcoaceticus*, *Clostridium species*, and *Salmonella typhimurium* in the gut of MS patients, we may suggest that GABAT enzymes from these bacterial species are involved in GABA degradation and the onset of MS. Although the gene encoding for GABAT is also present in gut bacteria of healthy individuals, including various strains of *Faecalibacterium* species, however, these microbes are not typically associated with MS. Aim of this study is to identify the variations in GABAT proteins that may contribute to its virulence in *Acinetobacter calcoaceticus*, *Clostridium species*, and *Salmonella typhimurium*, while being non-virulent in *Faecalibacterium* species.

## 2 Methodology

The methodology followed in present study is summarized in Fig. 1B.

### 2.1 Uniprot database

Sequences of GABAT protein in *Faecalibacterium* species, *Acinetobacter calcoaceticus, Clostridium* sp. and *Salmonella typhimurium* were retrieved from Uniprot database (https://www.uniprot.org, accessed on 13 January 2023) (Bairoch et al., 2005). Retrieved sequences alongwith accession IDs are shown in Supplementary data Table 1.

**Table 1:** Prediction of physicochemical properties of GABAT proteins documented in present study using Protparam tool.

#	Bacteria	No. of amino acids	Mol. Wt.	pI	GRAVY	Half life(hour)	Instability index	Aliphatic index
Bacteria from healthy individuals								
1	*Faecalibacterium* sp. An58	797	88057.47	5.67	−0.178	> 10	47.75	84.55
2	*Faecalibacterium* sp. An121	797	87957.30	5.48	−0.169	> 10	48.39	84.68
Bacteria from MS patients								
3	*Acinetobacter calcoaceticus* I	420	45286.25	6.06	0.070	> 10	38.23	99.52
4	*Acinetobacter calcoaceticus* II	446	49137.80	5.71	−0.235	> 10	32.57	88.79
5	*Acinetobacter calcoaceticus* III	428	45947.77	5.71	0.064	> 10	31.50	91.99
6	*Acinetobacter calcoaceticus* IV	447	48359.85	5.96	−0.125	> 10	30.56	89.55
7	*Acinetobacter calcoaceticus* V	430	45952.49	5.97	0.00	> 10	29.83	87.00
8	*Acinetobacter calcoaceticus* VI	447	48359.85	5.96	−0.125	> 10	30.56	89.55
10	*Clostridium* sp. I	450	49357.42	6.43	0.019	> 10	38.13	94.53
11	*Clostridium* sp. II	428	47177.46	6.04	−0.143	> 10	28.62	84.37
12	*Salmonella typhimurium*	427	45636.40	5.97	0.053	> 10	33.39	95.43

### 2.2 MEGA 11 software

To study the evolutionary relationship among the bacteria documented in present study with reference to GABAT protein, multiple sequence alignment was performed using CLUSTAL Omega Multiple Sequence Alignment tool (https://www.ebi.ac.uk/tools/msa/clustalo, accessed on 9 January 2024). Neighbor joining tree was constructed using the aligned sequences after gaps removal, using MEGA 11 software (https://www.megasoftware.net, accessed on 9 January 2024). The bootstrapping procedure with 100 bootstrap value was selected.

### 2.3 Protparam tool

Retrieved sequences were subjected to Protparam tool (https://web.expasy.org/protparam/, accessed on 19 January 2023) to analyze the physicochemical properties of GABAT protein in present study bacteria (Gasteiger et al., 2005). Properties computed include number of amino acids, molecular weight, isoelectric point (pI), grand average of hydropathy (GRAVY), half-life, instability index and aliphatic index.

### 2.4 Pepwheel program

To compute the hydrophilic, aliphatic and positively charged residues of GABAT proteins, Pepwheel program (https://bioinformatics.nl/cgi-bin/emboss/pepwheel, accessed on 20 January 2023) was consulted (Ramachandran G. N. and Sasisekharan, 1968). Results were obtained in the form of a helical wheel showing aliphatic, hydrophilic and positively charged residues as squares, diamonds and octagons, respectively.

### 2.5 SOSUI

To predict the hydropathy and charge plots SOSUI online tool (https://harrier.nagahama-i-bio.ac.jp/sosui/mobile/, accessed on 22 January 2023) was used (Mitaku and Sawada, 2016).

### 2.6 Gneg-mPLoc

All the bacteria addressed in present study are gram negative. For prediction of sub-cellular localization of GABAT proteins from these bacteria, Gneg-mPLoc server (https://www.csbio.sjtu.edu.cn/bioinf/Gneg-multi/, accessed on, 22 January 2023) was used (Shen and Chou, 2010).

### 2.7 PFP-FunDSeqE

To predict the functional domains in GABAT proteins, predicting protein fold pattern with functional domain and sequential evolution formation tool (PFP-FunDSeqE) (https://www.csbio.sjtu.edu.cn/bioinf/PFP-FunDSeqE/, accessed on 23 January 2023) was employed (Shen and Chou, 2009).

### 2.8 PROTEUS structure Prediction server

Secondary structures (2D) of bacterial GABAT proteins were assessed using PROTEUS Structure Prediction Server (https://www.proteus2.ca/proteus2/, 24 January 2023) (Montgomerie et al., 2006). Attributes of 2D structure computed were alpha helix, beta sheet and coil contents.

### 2.9 Alphafold and SAVES validation server

To predict the three dimensional (3D) configuration of GABAT proteins, AlphaFold Protein Structure Database (https://alphafold.ebi.ac.uk, 28 January 2023) was consulted (Ruff and Pappu, 2021). For validation of 3D structures, Procheck program of SAVES.6.0 structure validation server (https://saves.mbi.ucla.edu, accessed on 28 January 2023) was used (Laskowski et al., 2013).

### 2.10 B-cell epitopes prediction

To predict the B cell linear epitopes, Immune Epitope Database (IEDB) (tools.iedb.org/bcell/, accessed on 28 January 2023) was employed (Vita et al., 2019).

### 2.11 MEME suite 5.5.0

Conserved motifs in GABAT protein sequences were determined using MEME suite 5.5.0 (https://meme.sdsc.edu/meme/meme.html, 28 January 2023) (Bailey et al., 2009). For motifs prediction, by default values were used for all the parameters except the number of motifs. Ten motifs were predicted for each protein sequence.

### 2.12 HDOCK server

In order to check the best possible inhibitors of GABAT proteins from MS associated bacteria, binding affinities of proteins with Food and Drug Administration (FDA) approved GABAT inhibitors, HDOCK server (hdock.phys.hust.edu.cn, accessed on 28 January 2023) was used (Yan et al., 2020). Two anti-GABAT drugs, isoniazid and benzodiazepine were used for this analysis.

## 3 Results

### 3.1 Phylogeny of present study bacteria

**Fig. 2:**
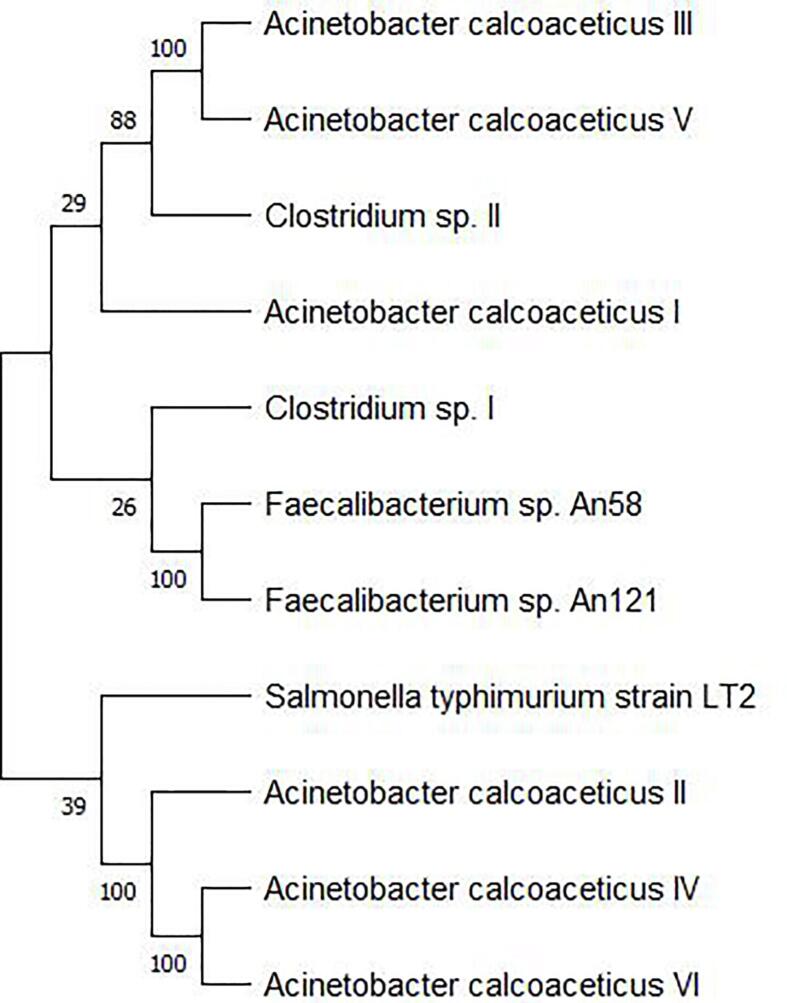
Phylogenetic tree constructed using MEGA 11 software to analyze the evolutionary relationship among the present study bacteria with reference to GABAT protein.

Phylogenetic tree is represented in (Fig. 2). According to this tree, *Faecalibacterium* sp. An58 and *Faecalibacterium* sp. An121 shared the same clade with bootstrap value of 100. *Acinetobacter calcoaceticus* III and V were more closely related to each other as compared to other. *Acinetobacter calcoaceticus* II, IV and IV originated from same branch point with bootstrap value of 100 so were more related. *Acinetobacter calcoaceticus* I and *Clostridium* sp. II were related with bootstrap value of 88. *Salmonella typhimurium* was found to be distantly related with other bacteria.

### 3.2 Prediction of physicochemical properties

Physicochemical properties of GABAT protein computed for present study bacteria are given in Table 1. The pI was observed in the range of 5.48–6.43. Highest value was observed in *Clostridium* sp. I and lowest in *Faecalibacterium* sp. An121. GRAVY was found to be ranging between −0.235 and 0.070 with the highest and lowest values recorded in *Acinetobacter calcoaceticus* II and *Acinetobacter calcoaceticus* I, respectively. Half-life was found same for all the bacterial GABAT proteins. Instability index was observed above 40 in *Faecalibacterium* sp. while below 40 in the bacteria associated with MS patients GIT. Highest value of aliphatic index was found in *Acinetobacter calcoaceticus* I and lowest in *Clostridium* sp. II. In all other bacteria, aliphatic index was intermediate between these values.

### 3.3 Prediction of hydrophilic, aliphatic and positively charged residues

Aliphatic, hydrophobic and positively charged residues content were found in the range of 32–44, 40–63 and 15–26, respectively (Supplementary data Fig. 1, Table 2). Number of aliphatic and hydrophilic residues were comparable between the bacteria from healthy individuals and MS patients gut. However, content of positively charged amino acids was lesser in strains of *Faecalibacterium* sp. than other bacteria documented in study.

**Table 2:** Prediction of sub-cellular localization, functional domains, aliphatic, hydrophilic and positively charged residues of GABAT protein in present study bacteria using Gneg-mPLoc, PFP-FunDSeqE and Pepwheel program, respectively.

**#**	Bacteria	Sub-cellular localization	Functional domain	Aliphatic residues	Hydrophilic residues	Positively charged residues
		Gneg-mPLoc	PFP-FunDSeqE	Pepwheel program		
Bacteria from healthy individuals						
1	*Faecalibacterium* sp. An58	Inner membrane, periplasm	4-helical cytokines	36	47	16
2	*Faecalibacterium* sp. An121	Inner membrane	4-helical cytokines	34	47	15
Bacteria from MS patients						
3	*Acinetobacter calcoaceticus* I	Cytoplasm	(TIM)-barrel	43	48	20
4	*Acinetobacter calcoaceticus* II	Cytoplasm	(TIM)-barrel	42	63	25
5	*Acinetobacter calcoaceticus* III	Cytoplasm	(TIM)-barrel	37	48	23
6	*Acinetobacter calcoaceticus* IV	Cytoplasm	(TIM)-barrel	43	56	22
7	*Acinetobacter calcoaceticus* V	Cytoplasm	(TIM)-barrel	32	49	25
8	*Acinetobacter calcoaceticus* VI	Cytoplasm	(TIM)-barrel	43	56	21
10	*Clostridium* sp. I	Cytoplasm	(TIM)-barrel	44	40	26
11	*Clostridium* sp. II	Cytoplasm	(TIM)-barrel	36	52	25
12	*Salmonella typhimurium*	Cytoplasm	(TIM)-barrel	44	44	26

### 3.4 Hydropathy plots prediction

The values of average of hydropathicity analyzed using SOSUI tool were same as predicted using Protparam. GABAT proteins from all the bacteria of present study were found to lack signal peptides (Supplementary data Fig. 2). The hydropathy values were recorded in the range of −2 to +2.

### 3.5 Sub-cellular localization

Sub-cellular localization analyses revealed that GABAT protein is located in inner-membrane of cell in strains of *Faecalibacterium* species as compared to GIT inhabiting bacteria of MS patients in which protein was found in cytoplasm (Table 2).

### 3.6 Functional domains prediction

Functional domains predicted using PFP-FunDSeqE revealed the presence of 4-helical cytokines in strains of *Faecalibacterium* sp. while (TIM)-barrel was found in all the MS patients GIT inhabiting bacteria (Table 2).

### 3.7 Two dimensional structure prediction

The 2D structure was predicted in terms of α-helix, β-sheet and coil contents (Supplementary data Fig. 3, Table 3). The α-helix was found to be the highest in strains of *Faecalibacterium* sp. i.e. 61 to 62 % as compared to the MS associated bacteria in which the values were observed in the range of 37 and 44. Beta sheet content was lowest in *Faecalibacterium* sp. strains (5 %) as compared to the value of 16–22 in other bacterial GABAT proteins. Coil content was comparable among the bacteria from healthy individuals and MS patients and was in the range of 33–45.

**Fig. 3:**
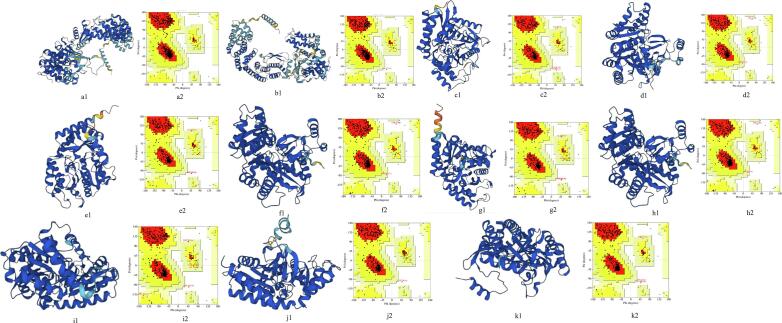
Three dimensional (3D) configuration of GABAT proteins of GIT bacteria of healthy individuals and MS patients and validation of these structures with the help of Ramachandran plots. (a1 and a2) *Faecalibacterium* sp. An58, (b1 and b2) *Faecalibacterium* sp. An121, (c1 and c2) *Acinetobacter calcoaceticus* I, (d1 and d2) *Acinetobacter calcoaceticus* II, (e1 and e2) *Acinetobacter calcoaceticus* III, (f1 and f2) *Acinetobacter calcoaceticus* IV, (g1 and g2) *Acinetobacter calcoaceticus* V, (h1 and h2) *Acinetobacter calcoaceticus* VI, (i1 and i2) *Clostridium* sp. I, (j1 and j2) *Clostridium* sp. II, (k1 and k2) *Salmonella typhimurium.*

**Table 3:** Prediction of 2D configuration of GABAT proteins in present study bacteria using PROTEUS Structure Prediction Server.

#	Bacteria	α-helix content % (no. of residues)	β-sheet content % (no. of residues)	Coil content % (no. of residues)
Bacteria from healthy individuals				
1	*Faecalibacterium* sp. An58	61 (4 8 7)	5 (36)	34 (2 7 4)
2	*Faecalibacterium* sp. An121	62 (4 9 6)	5 (36)	33 (2 6 5)
Bacteria from MS patients				
3	*Acinetobacter calcoaceticus* I	37 (1 5 5)	22 (91)	41 (1 7 4)
4	*Acinetobacter calcoaceticus* II	42 (1 7 9)	19 (83)	39 (1 6 6)
5	*Acinetobacter calcoaceticus* III	42 (1 8 2)	19 (81)	39 (1 6 7)
6	*Acinetobacter calcoaceticus* IV	38 (1 6 8)	18 (80)	45 (1 9 9)
7	*Acinetobacter calcoaceticus* V	46 (1 9 6)	16 (70)	38 (1 6 4)
8	*Acinetobacter calcoaceticus* VI	38 (1 7 0)	18 (82)	44 (1 9 5)
10	*Clostridium* sp. I	40 (1 8 0)	19 (84)	41 (1 8 6)
11	*Clostridium* sp. II	44 (1 8 8)	19 (80)	37 (1 6 0)
12	*Salmonella typhimurium*	44 (1 8 8)	17 (74)	39 (1 6 5)

### 3.8 Three dimensional structure prediction

The 3D structures were computed using Alphafold which showed that GABAT protein among the GIT bacteria of MS patients were significantly different in 3D form as compared to strains of *Faecalibacterium* sp (Fig. 3, Supplementary data Table 2). Although the structure also vary among strains of *Acinetobacter calcoaceticus*, *Clostridium* and *Salmonella* species but that variation was very small. Number of amino acid residues in allowed region were in the range of 88.4 to 95.1 showing stability of predicted protein models.

### 3.9 B-cell epitopes prediction

B-cell epitopes were found highest in two strains of *Faecalibacterium* sp. i.e. 18 and 19. On the other hand, in bacteria associated with MS, the epitopes were found in very small number i.e. *Acinetobacter calcoaceticus* I (9), *Acinetobacter calcoaceticus* II (6), *Acinetobacter calcoaceticus* III (4), *Acinetobacter calcoaceticus* IV and VI, *Clostridium* sp. I and II (5), *Acinetobacter calcoaceticus* V and *Salmonella typhimurium* (7) (Supplementary data Fig. 4, Supplementary data Table 3).

**Fig. 4:**
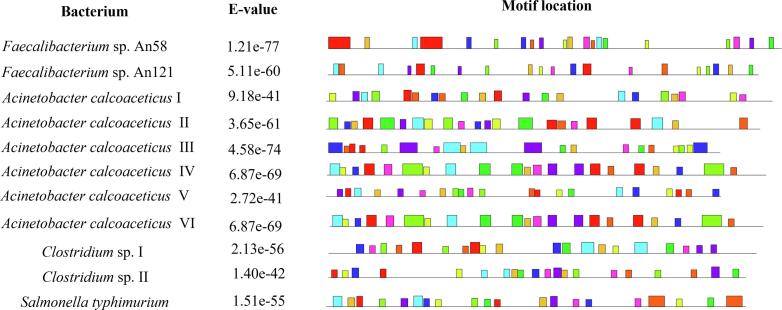
E-value and location of conserved protein motifs of GABAT proteins of GIT bacteria of healthy individuals and multiple sclerosis patients predicted using MEME suite.

### 3.10 Conserved domains

Total ten conserved motifs were predicted in GABAT proteins of present study bacteria (Fig. 4, Supplementary data Table 4). Among these bacteria, *Faecalibacterium* sp. An58 and An121 shared eight motifs i.e. NKDKNLIR, NDHYLW, SYFVPME, YRIRSDKT, FLYQTEFLGC, DFQAQC, DFSSLC and FYTAVW. *Acinetobacter calcoaceticus* II exhibited five conserved motifs same as found in *Acinetobacter calcoaceticus* IV and VI i.e. SYRLFYRNPV, RAMYMCTGS, RAMYMCTGS, RSTIMDTNSF and KLTDKR. *Acinetobacter calcoaceticus* III shared two conserved motifs i. e. MTMAMT and FLYPLT with *Acinetobacter calcoaceticus* V. Twenty protein motifs in *Acinetobacter calcoaceticus* IV were also present in *Acinetobacter calcoaceticus* VI i.e. SYRLFYRNPV**,** SFWGFGRHGV**,** YFNTFGGNPV**,** RAMYMCTGSE**,** RSTIMDTNSF**,** HCHPAVIEAVNAQMQMLNTH**,** TKQPDKALALNLIEELRNTH**,** IFIADEVQP**,** IFSSDGVMP**,** KVIQEE**,** KQIQQQ**,** YRVATDDLGEW**,** YHGTSDLTSGC**,** KLTDKR**,** WDAAGNKY**,** MNAKGIKF**,** KPMGNG**,** GPYGNV**,** RYLHEN and REEHAA.

**Table 4:** Scores reflecting binding affinities of GABAT proteins found in GIT bacteria of MS patients, with FDA approved drugs isoniazid and benzodiazepine, predicted using HDOCK server.

#	Bacteria	Score of GABAT docking with isoniazid(kcal/mol)	Score of GABAT docking with benzodiazepine(kcal/mol)
1	*Acinetobacter calcoaceticus* I	−213.15	−79.22
2	*Acinetobacter calcoaceticus* II	−240.60	−87.88
3	*Acinetobacter calcoaceticus* III	−229.30	−78.12
4	*Acinetobacter calcoaceticus* IV	−250.90	−87.88
5	*Acinetobacter calcoaceticus* V	−234.24	−85.08
6	*Acinetobacter calcoaceticus* VI	−250.90	−85.08
7	*Clostridium* sp. I	−267.42	−81.28
8	*Clostridium* sp. II	−244.22	−85.51
9	*Salmonella typhimurium*	–222.98	−83.23

### 3.11 Docking results

**Fig. 5:**
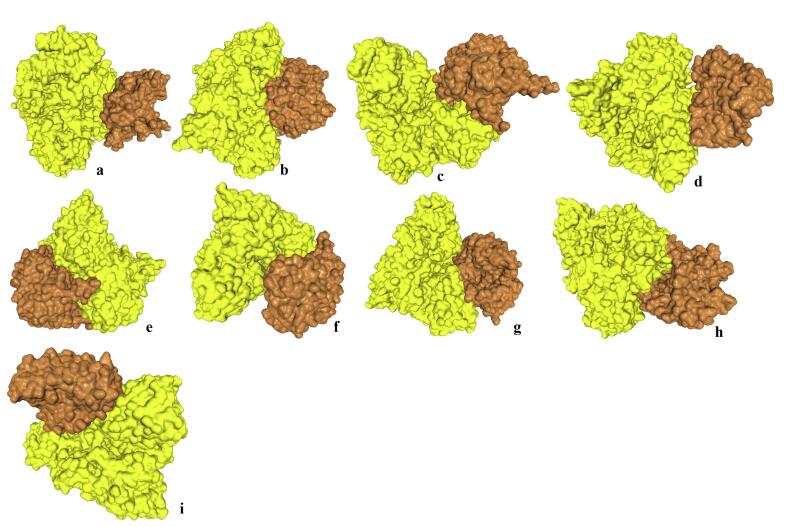
Docking analysis of GABAT proteins of GIT bacteria of multiple sclerosis patients with FDA approved drug isoniazid. (a) *Acinetobacter calcoaceticus* I, (b) *Acinetobacter calcoaceticus* II, (c) *Acinetobacter calcoaceticus* III, (d) *Acinetobacter calcoaceticus* IV, (e) *Acinetobacter calcoaceticus* V, (f) *Acinetobacter calcoaceticus* VI, (g) *Clostridium* sp. I, (h) *Clostridium* sp. II, (i) *Salmonella typhimurium.*

Docking analysis of GABAT proteins from MS associated bacteria with isoniazid and benzodiazepine revealed high binding affinities of protein for isoniazid with docking scores (-213.15 to −267.42) in all the bacteria as compared to benzodiazepine with docking scores (-78.12 to −87.88) (Fig. 5, Supplementary data Fig. 5, Table 4).

## 4 Discussion

The interference of gut associated bacteria in MS patients metabolism has been considerably explored and reported (Bhargava and Mowry, 2014, Cantarel et al., 2015, Cox et al., 2021, Durack and Lynch, 2019, Parker et al., 2020). Several of these studies also reported the role of GABAT in metabolic reduction of GABA in MS patients (Kowalczyk and Kulig, 2014, Nantes et al., 2017). To our knowledge, there has been no prior investigation focusing specifically on the characterization of GABAT in gut-inhabiting bacteria, particularly in the context of MS. Hence, this study represents first attempt to examine the various attributes of GABAT in these bacteria.

A study involving a control group of thirty-three healthy individuals and experimental group of twenty-two Chinese MS patients revealed the reduction in abundance of *Faecalibacterium* in MS effected individuals (Ling et al., 2020). A systematic review targeted the alpha and beta diversity among the MS patients by interpreting the literature published in ScienceDirect, Scopus, PubMed, Cochrane, CINAHL and Proquest databases and confirmed the reduction in proportion of *Faecalibacterium* (Ordoñez-Rodriguez et al., 2023). A study investigated the microbial composition of experimental group comprising of MS patients and control group comprising of healthy individuals. Both these groups contained seventy-one subjects. *Acinetobacter* was not only abundantly found in gut of MS patients but also observed to be associated with pro-inflammatory response in peripheral mononuclear blood cells (Cekanaviciute et al., 2017).

The pI is a measure of the pH at which a protein has a net charge of zero. It is a characteristic property of proteins and depends on the amino acid sequence of the protein. Activity of GABAT is influenced by the pH of its environment. Previous studies have shown that an alkaline pH is the optimal condition for this enzyme. (Bloch-Tardy et al., 1974). The pI values of GABAT in gut-associated bacteria of MS patients were found more alkaline (i.e., above pH 7) as compared to those in healthy individuals. Specifically, in *Acinetobacter calcoaceticus I*, *Clostridium* sp. *I and II* (which are associated with MS patients), the pI values of GABAT were higher as compared to the values of GABAT in other MS-associated and non-MS associated bacteria. This suggests that this protein would act as a more hydrophilic molecule at alkaline pH and the GABAT protein in the gut-associated bacteria of MS patients may behave differently at this pH, which may have implications for GABA degradation in MS (van Oss, 1997).

The half-life of all bacterial proteins was found to be longer, indicating their potential use as probiotics. This suggests that non-MS associated bacteria with less active GABAT enzymes have the potential to be used as probiotics to improve the gut microbiota of MS patients. Aliphatic index has direct relation with thermostability of globular proteins (Morya et al., 2012). Higher values were observed in majority of the diseased patients bacterial GABAT revealing their greater thermostability.

Instability index is a measure of in-vitro stability. The value below 40 shows greater protein stability (Gamage et al., 2019). In present study, GABAT from MS gut bacteria with instability index of 28.62 to 38.23 might be more stable in laboratory and can be easily manipulated through mutagenesis.

Analyses of conserved motifs revealed that the GABAT protein in healthy individuals' gut bacteria is not related to the same protein in bacteria inhabiting MS patients, as they do not share any of the motifs. However, both strains of *Faecalibacterium* species are related to each other, and *Acinetobacter calcoaceticus* II, III, IV, V, and VI are also related to some extent. *Salmonella typhimurium* and *Clostridium* species are also not related to any of the bacteria documented in the present study. The number of B-cell epitopes in a protein reflects its potential to stimulate the immune system. Current study found that the GABAT protein in non-MS associated bacteria had a greater number of epitopes as compared to MS-associated bacteria, indicating a greater potential for immune stimulation in the former group. This significant difference in epitope numbers may also explain the production of anti-inflammatory molecules in healthy individuals and the production of pro-inflammatory molecules in MS patients (Latvala, 2014). Additional experiments can be designed to examine the variations in immune-modulatory potential of GABAT proteins between individuals with and without the disease.

The differences observed in the functional domains, 2D and 3D configurations, conserved motifs, and B-cell epitopes of GABAT proteins between MS-associated and non-MS associated bacteria may be the underlying cause of its substantial involvement in MS pathology. These variations are justifying the strong link between dysbiosis and MS. Considering this, the MS can be treated significantly via replacing the gut bacteria of patients with the *Faecalibacterium* species.

## 5 Conclusion

Findings of this study could aid in elucidating the mechanisms behind MS pathogenesis caused by microbial dysbiosis. Currently, no diagnostic or prognostic biomarkers have been identified for MS. However, GABAT-producing gut bacteria targeted in present study i. e. *Acinetobacter calcoaceticus*, *Clostridium* sp. and *Salmonella typhimurium* could serve as potential prognostic biomarkers for MS in the future. Additionally, this study supports the idea of fecal microbiota transplantation (FMT) as a means of restoring gut microbiota and potentially reducing the abundance of GABA-degrading bacteria. Therefore, controlling GABA neurotransmission could be an effective strategy for protecting neurons in MS patients. In addition to this, GABAT protein of the MS patients gut associated bacteria can be mutated to enhance the number of immunogenic B-cell epitopes thus increasing its ability to stimulate the immune system.

Statements and Declarations:

**Funding:** N/A.

**Author contributions:** Nadia Hussain wrote the manuscript, Fatima Muccee perceived the idea, retrieved data from database, designed methodology and performed analysis.

**Submission declaration and verification:** The work is not published previously and it is not under consideration for publication elsewhere.

## CRediT authorship contribution statement

**Nadia Hussain:** Writing – original draft. **Fatima Muccee:** Conceptualization, Data curation, Methodology, Formal analysis.

## Declaration of Competing Interest

The authors declare that they have no known competing financial interests or personal relationships that could have appeared to influence the work reported in this paper.
