# Supplementary material for: In-silico characterization of GABAT protein found in gut-brain axis associated bacteria of healthy individuals and multiple sclerosis patients
Source: Saudi J Biol Sci. 2024 Feb 2;31(4):103939. doi: 10.1016/j.sjbs.2024.103939 (PMC10859293; doi:10.1016/j.sjbs.2024.103939)
Supplement: Supplementary Data 1 [file mmc1.docx]

**Supplementary data Table 1:** Sequences of GABAT proteins in bacteria documented in present study, retrieved from Uniprot database

| # | Bacterium | Sequence | Accession ID |
| --- | --- | --- | --- |
| 1 | [*Faecalibacterium* sp. An58](https://www.uniprot.org/taxonomy/1965648) | MIPFGWEDPVLYYPVRHHSPACAWHLQRVIRRYRPDCILIEGPEDAGPLLPLLADPLTQAPVALYCSYRDDTRLLETPEEGNDDPAAPVGTYACYYPFLDQSPELAAIRAAAALGVPAQFIDLPYAENLLATRAGRALRQEGRPENYLNDHYLWQNAFWQRVCEKTGMRSFEEFWEKYYETAGFAMSDEAFVRQMNAWCVLARQATPQEEMEADGCLAREARMAVRIAAAAARYQRVLVVAGGFHLWGLMHPRPEQTRPAVPADSQAVYPVRFSFEAADALSGYASGMPAPAFYTAVWQAAQAAPADAFDPAALWDQTVLGFLVKCGRRLRREGEVLSAFDEMCAMQQARGLAALRDKKAPGLYEMQDAVLSSFVKGEANLSGCQPLRVLRQLTTGNGVGQLAPNSLVPPLVQDFQAQCRRYRIRSDKTSLQELTLEIFAKPAHRGASRFLYQTEFLGCGFARRTKGPDLRRNKDKNLIRESWEYRWTASVEAALVERSVAGSTVREACARELRQRMARAGRAGEGADLLLQGFLMGIGEDAGSFARRLEELLLGDGDFSSLCQACRALASLEEWQVQYGEAGRCDAAGLLGRCFDRILQLLPAMGAADDRGAPEVQRCCLLLYQITQRDAFAGMRPRLLAALEELAGQNPVQPALHGTALGLLYGADPGWKARIDTALRGYLQGTRGMMLASAAFLQGLFYTAKDLLLVDPGFLKQVDGLLCALSDQDFDALLPELRLAFSYFVPMETDRIARQAAALHRGAAGTLRQAGISPAEYSRNEQVDAWAAARLAEEAIP | A0A1Y3WRL8 |
| 2 | [*Faecalibacterium* sp. An121](https://www.uniprot.org/taxonomy/1965550) | MIPFGWEDPVLYYPVRHHSPACAWHLQRVIQRYRPECILIEGPEDAGPLLPLLADPLTQAPVALYCSYRDDARLLETPEEGNDDPAAPVGTYACYYPFLDQSPELAAIRAAAALGVPAQFIDLPYAENLLATRAGRALRQEGRPENYLNDHYLWQNAFWQRVCEKTGMRSFEEFWEKYYETAGFAMSDEAFVRQMNAWCVLARQATPQEEMEADGCLAREARMAVRIAAAAARYQRVLVVAGGFHLWGLMHPRPEQTRPAVPADSQAVYPVRFSFEAADALSGYASGMPAPAFYTAVWQAAQAAPADAFDPAALWDETVLGFLVKCGRRLRREGEVLSAFDEMCAMQQARGLAALRDKRAPGLYEMQDAVLSSFVKGEANLSGCQPLRVLRQLTTGNGVGQLAPNSLVPPLVQDFQAQCRRYRIRSDKTSLQELTLEIFAKPAHRGASRFLYQTEFLGCGFARRTKGPDLRRNKDKNLIRESWEYRWTASVEAALVERSVAGSTVREACARELRQRMAGAGRAGEGADLLLQGFLMGIGEDAGSFARRLEELLLGDGDFSSLCQACRALASLEEWQVQYGEAGRCDAAGLLGRCFDRILQLLPAMGAADDRGAPEVQRCCLLLYQITQREAFAGMRPRLLAALEELAGQNPVQPALHGTALGLLYGADPGWKARIDTALRGYLQGTRGMMLASAAFLQGLFYTAKDLLLVDPGFLKQVDGLLCALSDQDFDALLPELRLAFSYFVPMETDRIARQAAALHRGAAGTLRQAGISPAEYSRNEQVDAWAAARLAEEAIP | A0A1Y4T8V7 |
| Multiple sclerosis | | | |
| 1 | [*Acinetobacter calcoaceticus*](https://www.uniprot.org/taxonomy/471)  I | MNNSQGISKALGLIHPITIQKGLNAEVWDEQGKRYIDFVGGIGVLNFGHCNPSIVTAIQKQAETLTHYAFNAAMHRPYLSFMEKLLKIIPISEPLAGMFTNSGAEATENALKVARIVTKRTAVIAFDGGFHGRTLAAVNLNGKTRPYKDGLGPLPGPVYHIPYPSADNGVTVEQAQAALQRLIQVEVDVEEIAAFIFEPVLGEGGFHIMQPAFAQYLRQFCDQHGILIIADEIQSGFARTGHDFAIRHLGIEPDLMLLGKSIAGGLPLGALVGRAKYMDFPTKGALGGTYSGNPIACAAGLATLDILQSDQFQASVQHYIQCIEQRYAKWQQMQLTPWLGRLTGVGAMRGIELQHPELGAGTAQLAQVMADAREQGLLLMSSGPAKNIIRLLTPLTISPEMLDEGLDILEAVLAKTATAQ | A0A4V2R0Y5 |
| 2 | [*Acinetobacter calcoaceticus*](https://www.uniprot.org/taxonomy/471)  II | MGTRSTIMDTNSFRPEHAAELDHATRKLTDKRNKILGESYRLFYRNPVHLVKGLGQYLWDAAGHQYLDVYNNVASIGHCHPAVIEAVNEQMKMLNTHTRYLHENILDYTEELLQTTPDEIDRAMYMCTGSEANDLAIRIAREYSGGTGIIVSQEAYHGTSSLTSGCSPALGTEQRLDDTTRLVPAPDYYRIQTDDLGEWFAQQIQQQIDDMNAKGIKFAGFLADSIFSSDGVMPNPVGFLQKAVDVVHANGGIFIADEVQPGFARTGDSFWGFGRHNVVPDIITTGKPMGNGVPVSGLLAKSEVLAAFSDKIPYFNTFGGNPVSMAAAQAVLKVIQEEELQTHSKYVGGLLLKELTKLMDKHEVVGDVRGAGLFIGFELVKDRGTKEPNKALALNLIEELRNTHRVLTSVAGPYGNVLKLRPPLAFQTKDIDWLVGALDQALTKLK | A0A1A0GDL6 |
| 3 | [*Acinetobacter calcoaceticus*](https://www.uniprot.org/taxonomy/471)  III | MDNQYSALNARKQQSTPRGVGVMCQWYVERAENATLWDQEGREFIDFAGGIAVLNTGHCHPKIMAAVTEQLGKFTHTAYQVVPYESYISLVERLIERAPIAGPAKAALFTTGAEAVENAIKIARAATKRHGVITFSGAFHGRSFMTMAMTGKTAPYKRDFGVMPGGVFHARYPVAINNVSVDDAIESLEEIFSADIAAHDVAAIVLEPVQGEGGFNVAPQALMIRLRQICDEHGILLIADEVQTGFARTGKLFAMDYHPEAKPDLMTMAKSLGGGFPISAVVGRAEVMDAPAPGGLGGTYAGNPLAVAAAHAVLDVIEEEKLCERANKLGAELVNVLEDLKDSHSFVRDIRGIGSMIAVELDTAERAKAIQDDAMSKNLILLTCGKKANVIRFLYPLTIPDAQFQAALAILKQSFDATSALKNMGVTA | A0A4R1XU87 |
| 4 | [*Acinetobacter calcoaceticus*](https://www.uniprot.org/taxonomy/471)  IV | MVTRSTIMDTNSFREEHAAALDADTRKLTDKRAQLLGESYRLFYRNPVHLVKGQGQYLWDAAGNKYLDVYNNVASIGHCHPAVIEAVNAQMQMLNTHTRYLHENILDYSEALLATAPAEIDRAMYMCTGSEANDLAIRIARSFSGGTGIIVSQEAYHGTSDLTSGCSPALGSGQALPATTRLVPAPDQYRVATDDLGEWFAKQIQQQIDDMNAKGIKFAGFLADSIFSSDGVMPNPVGYLQKAVDVVHANGGIFIADEVQPGFARTGDSFWGFGRHGVVPDIITTGKPMGNGIPVSGLLAKSHVLAAFSDHIPYFNTFGGNPVAMAAAQAVLKVIQEEQLQAHSKKVGAQLQAELSKLMDRHPRIGDVRGAGLFIGFELVTDRDTKQPDKALALNLIEELRNTHRVLTSVAGPYGNVLKLRPPLAFQASDIDWLVGALDQSLSKLGA | A0A4R1Y3Y8 |
| 5 | [*Acinetobacter calcoaceticus*](https://www.uniprot.org/taxonomy/471)  V | MDNQHSALNARKQQATPRGVGVMCQWYAEKAENATLWDKEGNQFIDFAGGIAVLNTGHRHPKVIAAVTEQLTKFTHTAYQVTPYESYVALAERINERAPIAGPAKSAFFTTGAEAVENAVKIARCYTGRHGIITFGNGFHGRSFMTMAMTGKTAPYKRDFGVMPAGVFHARYPVPAKGISVDAAIESVEDIFSEDIAPHDVAAIVLEPVQGEGGFHVVPAEFLKRLRAICDKHGILLVADEVQSGFARTGKLFAMNHYETKADLITMAKSLGGGFPISGVVGRAEVMDAPNPGGLGGTYAGSPIAVAAAHAVIDAIEEENLCDRANELGAELVVSLKDIQQATGNVVTDIRALGSMVAVELETAEQAKVVQNYAMENGLLLLTCGKYGNVIRFLYPLTIPAEQFRQGLDILKQGFAALKAGSAKAMEQSA | A0A446ZF26 |
| 6 | [*Acinetobacter calcoaceticus*](https://www.uniprot.org/taxonomy/471)  VI | MVTRSTIMDTNSFREEHAAALDADTRKLTDKRAQLLGESYRLFYRNPVHLVKGQGQYLWDAAGNKYLDVYNNVASIGHCHPAVIEAVNAQMQMLNTHTRYLHENILDYSEALLATAPAEIDRAMYMCTGSEANDLAIRIARSFSGGTGIIVSQEAYHGTSDLTSGCSPALGSGQALPATTRLVPAPDQYRVATDDLGEWFAKQIQQQIDDMNAKGIKFAGFLADSIFSSDGVMPNPVGYLQKAVDVVHANGGIFIADEVQPGFARTGDSFWGFGRHGVVPDIITTGKPMGNGIPVSGLLAKSHVLAAFSDHIPYFNTFGGNPVAMAAAQAVLKVIQEEQLQAHSKKVGAQLQAELSKLMDRHPRIGDVRGAGLFIGFELVTDRDTKQPDKALALNLIEELRNTHRVLTSVAGPYGNVLKLRPPLAFQASDIDWLVGALDQSLSKLGA | A0A4R1Y3Y8 |
| 7 | [*Clostridium* sp](https://www.uniprot.org/taxonomy/59620).  I | MLKNELPRIVTKQIPGPKAAKWIARRGEAVPSAIKCVYPIVIARGEGAMLEDVDGNYFLDWVGGVGVLNIGYSRPELIEAVKKQSERYFHGMFNIVTHEGYVALAEKLNAIAPVKGTKRKTYFANSGAEADENAVKIAKAFTKRPNIIVFSGAFHGRTMLTMAMTSKKAYAYGMGPFPDGVYRAEFPYLYRKLEGIPETEAISYYIKSIEQVFEQASPAEFVAAIVVEPLQGEGGFVPAPIEWVKAVRKICDKYGILLVADEVQSGFCRTGRMFASDYWKDADAQPDIVTTAKSIAGGIPLSAITAREEIMEAVPCGVIGGTFCGNPLACASALQVIEIMEKENFEARAVEIEKKVMKRYQKMQKKLPVIGDVRGIGAMIGLEIVKDQESKQPNPTLVSEVVQECIQNGLIIENAGIYGNVIRFLAPLVITDEQLEAGLDILEAAIRRCM | A0A1C5R0E2 |
| 8 | [*Clostridium* sp](https://www.uniprot.org/taxonomy/59620).  II | MTQHERSKVLLPPMAMHAETTPCVVRGEGCYLYTEDGRKILDMASGIATNALGHCHPKVIAAAEKQLHTLIHAGHNILYYEPYTTLMEKLVEKTGNKYKVYFSNSGAEANEGAMKLAKYATQRPVIISMKNAFHGRTMATATITTSNAAYRKNYEPMMPSVYFAEYPYLFRTPYKMEDGKCPKEYFTQFDEIFKKIVDPSMVAAIMMEPVQGEGGYVVPPVEWLKYVRELCDKHGIMLIFDEVQTGFGRTGNLYAWQTLGVEPDIFTSAKAIAGGLPLSAVFGKKEIMDKWAKGAHGGTYGGNPVSCAASLAVLEELYEGGVLENVKKMGEVVRGKFYDLQKKYDVIGDVRGLGLMNAIEFVKPEDNAPDGALCAAVQAEALKRDLLLLNCGADHNNIRLIPPLNVDEATLDTVFQIIDESIAAALKG | A0A1C5T457 |
| 9 | [*Salmonella typhimurium* (strain LT2 / SGSC1412 / ATCC 700720)](https://www.uniprot.org/taxonomy/99287)  i | MNTNNALMQRRHNAVPRGVGQIHPIFAERAENCRVWDVEGREYLDFAGGIAVLNTGHLHPGIVSAVEAQLKKLSHTCFQVLAYEPYLALCERMNQKVPGDFAKKTLLVTTGSEAVENAVKIARAATKRSGAIAFSGAYHGRTHYTLSLTGKVHPYSAGMGLMPGHVYRALYPCPLHNISDDDAIASIERIFKNDAAPEDIAAIIIEPVQGEGGFYAASPAFMQRLRALCDQHGIMLIADEVQSGAGRTGTLFAMEQMGVAADITTFAKSIAGGFPLAGVTGRADVMDAIAPGGLGGTYAGNPIACAAALAVLDIFEQENLLQKANTLGKTLRDGLMEIAETHREIGDVRGLGAMIAIELFENGDPGKPNAALTADIVTRAREKGLILLSCGPYYNILRILVPLTIEASQIRQGLEIIAQCFDEAKQA | Q8ZMM1 |

**Supplementary data Table 2:** Validation of 3D structures of GABAT proteins found in GIT bacteria of normal individuals and MS patients, using Procheck program of SAVES.6.0 structure validation server

| # | Bacteria | Residues in most favored region | Residues in additional allowed regions | Residues in disallowed region | G-factor |
| --- | --- | --- | --- | --- | --- |
| 1 | [*Faecalibacterium* sp. An58](https://www.uniprot.org/taxonomy/1965648) | 95.1 | 4.9 | 0.0 | 0.21 |
| 2 | [*Faecalibacterium* sp. An121](https://www.uniprot.org/taxonomy/1965550) | 94.8 | 5.1 | 0.1 | 0.21 |
| 3 | [*Acinetobacter calcoaceticus*](https://www.uniprot.org/taxonomy/471) I | 91.8 | 7.4 | 0.6 | 0.11 |
| 4 | [*Acinetobacter calcoaceticus*](https://www.uniprot.org/taxonomy/471) II | 88.6 | 10.9 | 0.3 | 0.10 |
| 5 | [*Acinetobacter calcoaceticus*](https://www.uniprot.org/taxonomy/471) III | 89.8 | 9.4 | 0.5 | 0.10 |
| 6 | [*Acinetobacter calcoaceticus*](https://www.uniprot.org/taxonomy/471) IV | 88.8 | 10.6 | 0.3 | 0.09 |
| 7 | [*Acinetobacter calcoaceticus*](https://www.uniprot.org/taxonomy/471) V | 90.5 | 8.4 | 0.5 | 0.09 |
| 8 | [*Acinetobacter calcoaceticus*](https://www.uniprot.org/taxonomy/471) VI | 88.8 | 10.6 | 0.3 | 0.09 |
| 10 | [*Clostridium* sp](https://www.uniprot.org/taxonomy/59620). I | 90.6 | 8.3 | 0.3 | 0.08 |
| 11 | [*Clostridium* sp](https://www.uniprot.org/taxonomy/59620). II | 89.4 | 10.3 | 0.3 | 0.07 |
| 12 | *Salmonella typhimurium* | 88.4 | 0.7 | 0.3 | 0.10 |

**Supplementary data Table 3:** B-cell epitopes in GABAT proteins found in GIT bacteria of normal individuals and MS patients, predicted using Immune Epitope Database (IEDB)

| # | Start | End | Peptide sequence |
| --- | --- | --- | --- |
| [*Faecalibacterium* sp. An58](https://www.uniprot.org/taxonomy/1965648) | | | |
| 1 | 71 | 89 | DTRLLETPEEGNDDPAAPV |
| 2 | 132 | 157 | TRAGRALRQEGRPENYLNDHYLWQNA |
| 3 | 167 | 190 | GMRSFEEFWEKYYETAGFAMSDEA |
| 4 | 205 | 216 | ATPQEEMEADGC |
| 5 | 251 | 263 | HPRPEQTRPAVPA |
| 6 | 280 | 288 | ALSGYASGM |
| 7 | 302 | 317 | QAAPADAFDPAALWDQ |
| 8 | 358 | 364 | KKAPGLY |
| 9 | 378 | 385 | EANLSGCQ |
| 10 | 394 | 403 | TTGNGVGQLA |
| 11 | 420 | 445 | RRYRIRSDKTSLQELTLEIFAKPAHR |
| 12 | 462 | 482 | ARRTKGPDLRRNKDKNLIRES |
| 13 | 551 | 558 | ELLLGDGD |
| 14 | 575 | 586 | WQVQYGEAGRCD |
| 15 | 607 | 614 | AADDRGAP |
| 16 | 628 | 635 | QRDAFAGM |
| 17 | 742 | 751 | SYFVPMETDR |
| 18 | 758 | 786 | ALHRGAAGTLRQAGISPAEYSRNEQVDAW |
| [*Faecalibacterium* sp. An121](https://www.uniprot.org/taxonomy/1965550) | | | |
| 1 | 71 | 89 | DARLLETPEEGNDDPAAPV |
| 2 | 131 | 157 | ATRAGRALRQEGRPENYLNDHYLWQNA |
| 3 | 167 | 190 | GMRSFEEFWEKYYETAGFAMSDEA |
| 4 | 205 | 218 | ATPQEEMEADGCLA |
| 5 | 250 | 263 | MHPRPEQTRPAVPA |
| 6 | 280 | 288 | ALSGYASGM |
| 7 | 302 | 316 | QAAPADAFDPAALWD |
| 8 | 358 | 363 | KRAPGL |
| 9 | 378 | 385 | EANLSGCQ |
| 10 | 394 | 403 | TTGNGVGQLA |
| 11 | 420 | 445 | RRYRIRSDKTSLQELTLEIFAKPAHR |
| 12 | 462 | 482 | ARRTKGPDLRRNKDKNLIRES |
| 13 | 551 | 558 | ELLLGDGD |
| 14 | 575 | 586 | WQVQYGEAGRCD |
| 15 | 606 | 614 | GAADDRGAP |
| 16 | 629 | 635 | REAFAGM |
| 17 | 684 | 689 | QGTRGM |
| 18 | 742 | 751 | SYFVPMETDR |
| 19 | 758 | 786 | ALHRGAAGTLRQAGISPAEYSRNEQVDAW |
| [*Acinetobacter calcoaceticus*](https://www.uniprot.org/taxonomy/471) I | | | |
| 1 | 5 | 14 | QGISKALGLI |
| 2 | 87 | 92 | KIIPIS |
| 3 | 143 | 153 | KTRPYKDGLGP |
| 4 | 166 | 175 | ADNGVTVEQA |
| 5 | 183 | 189 | IQVEVDV |
| 6 | 278 | 285 | MDFPTKGA |
| 7 | 317 | 322 | QHYIQC |
| 8 | 325 | 336 | QRYAKWQQMQLT |
| 9 | 356 | 363 | PELGAGTA |
| [*Acinetobacter calcoaceticus*](https://www.uniprot.org/taxonomy/471) II | | | |
| 1 | 5 | 45 | STIMDTNSFRPEHAAELDHATRKLTDKRNKILGESYRLFYR |
| 2 | 170 | 178 | LGTEQRLDD |
| 3 | 189 | 203 | YRIQTDDLGEWFAQQ |
| 4 | 205 | 215 | QQQIDDMNAKG |
| 5 | 382 | 390 | DRGTKEPNK |
| 6 | 428 | 433 | TKDIDW |
| [*Acinetobacter calcoaceticus*](https://www.uniprot.org/taxonomy/471) III | | | |
| 1 | 7 | 21 | ALNARKQQSTPRGVG |
| 2 | 153 | 161 | TAPYKRDFG |
| 3 | 175 | 186 | AINNVSVDDAIE |
| 4 | 288 | 294 | MDAPAPG |
| [*Acinetobacter calcoaceticus*](https://www.uniprot.org/taxonomy/471) IV | | | |
| 1 | 5 | 45 | STIMDTNSFREEHAAALDADTRKLTDKRAQLLGESYRLFYR |
| 2 | 170 | 175 | LGSGQA |
| 3 | 189 | 203 | YRVATDDLGEWFAKQ |
| 4 | 205 | 215 | QQQIDDMNAKG |
| 5 | 382 | 391 | DRDTKQPDKA |
| [*Acinetobacter calcoaceticus*](https://www.uniprot.org/taxonomy/471) V | | | |
| 1 | 5 | 22 | HSALNARKQQATPRGVGV |
| 2 | 153 | 161 | TAPYKRDFG |
| 3 | 175 | 183 | PAKGISVDA |
| 4 | 192 | 197 | FSEDIA |
| 5 | 287 | 295 | MDAPNPGGL |
| 6 | 320 | 325 | NLCDRA |
| 7 | 401 | 406 | AEQFRQ |
| [*Acinetobacter calcoaceticus*](https://www.uniprot.org/taxonomy/471) VI | | | |
| 1 | 5 | 45 | STIMDTNSFREEHAAALDADTRKLTDKRAQLLGESYRLFYR |
| 2 | 170 | 175 | LGSGQA |
| 3 | 189 | 203 | YRVATDDLGEWFAKQ |
| 4 | 205 | 215 | QQQIDDMNAKG |
| 5 | 382 | 391 | DRDTKQPDKA |
| [*Acinetobacter calcoaceticus*](https://www.uniprot.org/taxonomy/471) VII | | | |
| 1 | 5 | 35 | ELPRIVTKQIPGPKAAKWIARRGEAVPSAIK |
| 2 | 168 | 176 | KAYAYGMGP |
| 3 | 190 | 201 | YRKLEGIPETEA |
| 4 | 351 | 359 | EIEKKVMKR |
| 5 | 387 | 396 | DQESKQPNPT |
| [*Clostridium* sp](https://www.uniprot.org/taxonomy/59620). I | | | |
| 1 | 5 | 35 | ELPRIVTKQIPGPKAAKWIARRGEAVPSAIK |
| 2 | 168 | 176 | KAYAYGMGP |
| 3 | 190 | 201 | YRKLEGIPETEA |
| 4 | 351 | 359 | EIEKKVMKR |
| 5 | 387 | 396 | DQESKQPNPT |
| [*Clostridium* sp](https://www.uniprot.org/taxonomy/59620). II | | | |
| 1 | 147 | 156 | NAAYRKNYEP |
| 2 | 171 | 182 | RTPYKMEDGKCP |
| 3 | 190 | 198 | DEIFKKIVD |
| 4 | 288 | 296 | MDKWAKGAH |
| 5 | 364 | 371 | PEDNAPDG |
| *Salmonella typhimurium* | | | |
| 1 | 6 | 22 | ALMQRRHNAVPRGVGQI |
| 2 | 95 | 100 | QKVPGD |
| 3 | 152 | 161 | VHPYSAGMGL |
| 4 | 173 | 182 | CPLHNISDDD |
| 5 | 192 | 197 | KNDAAP |
| 6 | 286 | 292 | MDAIAPG |
| 7 | 362 | 370 | MDAIAPG |

**Supplementary data Table 4:** Conserved motifs prediction in GABAT proteins found in GIT bacteria of normal individuals and MS patients, using MEME suite 5.5.0

| # | Bacterium | E-value | p-value | Motif sites |
| --- | --- | --- | --- | --- |
| 1 | [*Faecalibacterium* sp. An58](https://www.uniprot.org/taxonomy/1965648) | 2.9e+000 | 4.22e-39 | **LWQNAFWQRV CEKTGMRSFEEFWEKYYETAGFAMSDEAFVRQMNAWCV LARQATPQEE** |
|  |  |  | 8.35e-39 | **MIPFGWEDPVLYYPVRHHSPACAWHLQRVIRRYRPDCI LIEGPEDAGP** |
|  |  | 2.8e+001 | 1.27e-10 | **RRTKGPDLRR NKDKNLIR ESWEYRWTAS** |
|  |  |  | 1.64e-10 | **RQEGRPENYL NDHYLWQN AFWQRVCEKT** |
|  |  | 3.8e+001 | 8.44e-9 | **GYASGMPAPA FYTAVW QAAQAAPADA** |
|  |  |  | 1.53e-8 | **LASAAFLQGL FYTAKD LLLVDPGFLK** |
|  |  | 8.6e+001 | 5.78e-10 | **GLYEMQDAVL SSFVKGE ANLSGCQPLR** |
|  |  |  | 8.37e-10 | **ALLPELRLAF SYFVPME TDRIARQAAA** |
|  |  | 1.6e+002 | 9.04e-11 | **VQDFQAQCRR YRIRSDKT SLQELTLEIF** |
|  |  |  | 1.73e-10 | **DPLTQAPVAL YCSYRDDT RLLETPEEGN** |
|  |  | 9.4e+002 | 1.99e-10 | **TLRQAGISPA EYSRNEQV DAWAAARLAE** |
|  |  |  | 1.53e-9 | **KDKNLIRESW EYRWTASV EAALVERSVA** |
|  |  | 4.5e+001 | 1.86e-10 | **YQRVLVVAGG FHLWGLMH PRPEQTRPAV** |
|  |  |  | 2.53e-10 | **LRREGEVLSA FDEMCAMQ QARGLAALRD** |
|  |  | 9.8e+002 | 4.09e-12 | **AKDLLLVDPG FLKQVDGLLC ALSDQDFDAL** |
|  |  |  | 5.69e-12 | **AKPAHRGASR FLYQTEFLGC GFARRTKGPD** |
|  |  | 1.0e+003 | 3.58e-8 | **MQQARGLAAL RDKKAP GLYEMQDAVL** |
|  |  |  | 1.17e-7 | **QTEFLGCGFA RRTKGP DLRRNKDKNL** |
|  |  | 1.0e+003 | 4.03e-8 | **PNSLVPPLVQ DFQAQC RRYRIRSDKT** |
|  |  |  | 8.70e-8 | **RLEELLLGDG DFSSLC QACRALASLE** |
| 2 | [*Faecalibacterium* sp. An121](https://www.uniprot.org/taxonomy/1965550) | 1.1e+000 | 7.19e-19 | **RRTKGPDLRR NKDKNLIRESWEYRW TASVEAALVE** |
|  |  |  | 1.45e-17 | **QNAFWQRVCE KTGMRSFEEFWEKYY ETAGFAMSDE** |
|  |  | 5.6e+000 | 4.94e-13 | **IPFGWEDPVL YYPVRHHSP ACAWHLQRVI** |
|  |  |  | 1.22e-11 | **PAAPVGTYAC YYPFLDQSP ELAAIRAAAA** |
|  |  | 3.1e+001 | 8.01e-9 | **GYASGMPAPA FYTAVW QAAQAAPADA** |
|  |  |  | 1.39e-8 | **LASAAFLQGL FYTAKD LLLVDPGFLK** |
|  |  | 9.5e+001 | 6.46e-9 | **RQEGRPENYL NDHYLW QNAFWQRVCE** |
|  |  |  | 2.04e-8 | **ARYQRVLVVA GGFHLW GLMHPRPEQT** |
|  |  | 9.8e+001 | 5.76e-10 | **GLYEMQDAVL SSFVKGE ANLSGCQPLR** |
|  |  |  | 9.49e-10 | **ALLPELRLAF SYFVPME TDRIARQAAA** |
|  |  | 3.3e+002 | 1.48e-9 | **AGISPAEYSR NEQVDAW AAARLAEEAI** |
|  |  |  | 4.47e-9 | **AGFAMSDEAF VRQMNAW CVLARQATPQ** |
|  |  | 8.7e+002 | 4.00e-12 | **AKDLLLVDPG FLKQVDGLLC ALSDQDFDAL** |
|  |  |  | 5.49e-12 | **AKPAHRGASR FLYQTEFLGC GFARRTKGPD** |
|  |  | 9.5e+002 | 3.86e-8 | **PNSLVPPLVQ DFQAQC** **RRYRIRSDKT** |
|  |  |  | 8.69e-8 | **RLEELLLGDG DFSSLC QACRALASLE** |
|  |  | 1.2e+003 | 1.55e-12 | **YYPVRHHSPA CAWHLQRVIQ RYRPECILIE** |
|  |  |  | 7.07e-11 | **DDRGAPEVQR CCLLLYQITQ REAFAGMRPR** |
|  |  | 8.7e+002 | 4.17e-8 | **SGCQPLRVLR QLTTGN GVGQLAPNSL** |
|  |  |  | 6.73e-8 | **RIDTALRGYL QGTRGM MLASAAFLQG** |
| 3 | [*Acinetobacter calcoaceticus*](https://www.uniprot.org/taxonomy/471)  I | 8.1e-001 | 3.21e-10 | **TLTHYAFNAA MHRPYLS FMEKLLKIIP** |
|  |  |  | 1.09e-9 | **DGLGPLPGPV YHIPYPS ADNGVTVEQA** |
|  |  | 1.3e+001 | 2.17e-9 | **LPLGALVGRA KYMDFP TKGALGGTYS** |
|  |  |  | 3.18e-8 | **NAEVWDEQGK RYIDFV GGIGVLNFGH** |
|  |  | 2.9e+001 | 2.19e-9 | **RYIDFVGGIG VLNFGHC NPSIVTAIQK** |
|  |  |  | 2.69e-9 | **ILQSDQFQAS VQHYIQC IEQRYAKWQQ** |
|  |  | 6.7e+001 | 4.24e-9 | **PITIQKGLNA EVWDEQ GKRYIDFVGG** |
|  |  |  | 7.68e-8 | **QAALQRLIQV EVDVEE IAAFIFEPVL** |
|  |  | 3.9e+001 | 9.87e-8 | **LAAVNLNGKT RPYKDG LGPLPGPVYH** |
|  |  |  | 2.45e-7 | **IMQPAFAQYL RQFCDQ HGILIIADEI** |
|  |  |  | 7.62e-7 | **VQHYIQCIEQ RYAKWQ QMQLTPWLGR** |
|  |  | 4.2e+002 | 4.81e-8 | **TKRTAVIAFD GGFHGR TLAAVNLNGK** |
|  |  |  | 1.49e-7 | **AFIFEPVLGE GGFHIM QPAFAQYLRQ** |
|  |  | 7.8e+002 | 2.81e-8 | **DFPTKGALGG TYSGNP IACAAGLATL** |
|  |  |  | 1.23e-7 | **PISEPLAGMF TNSGAE ATENALKVAR** |
|  |  | 1.0e+003 | 9.61e-9 | **IEQRYAKWQQ MQLTPW LGRLTGVGAM** |
|  |  |  | 2.38e-6 | **MSSGPAKNII RLLTPL TISPEMLDEG** |
|  |  | 1.7e+003 | 5.36e-8 | **AAMHRPYLSF MEKLLK IIPISEPLAG** |
|  |  |  | 2.96e-7 | **GMFTNSGAEA TENALK VARIVTKRTA** |
|  |  | 2.2e+003 | 7.26e-8 | **DAREQGLLLM SSGPAK NIIRLLTPLT** |
|  |  |  | 1.57e-7 | **MNN SQGISK ALGLIHPITI** |
| 4 | [*Acinetobacter calcoaceticus*](https://www.uniprot.org/taxonomy/471)  II | 2.2e+000 | 3.71e-11 | **TDKRNKILGE SYRLFYRNPV HLVKGLGQYL** |
|  |  |  | 9.86e-11 | **VQPGFARTGD SFWGFGRHNV VPDIITTGKP** |
|  |  |  | 4.03e-10 | **VLAAFSDKIP YFNTFGGNPV SMAAAQAVLK** |
|  |  |  | 9.21e-9 | **FAGFLADSIF SSDGVMPNPV GFLQKAVDVV** |
|  |  | 1.3e+001 | 4.39e-14 | **AAAQAVLKVI QEEELQTHSKY VGGLLLKELT** |
|  |  |  | 1.48e-13 | **HPAVIEAVNE QMKMLNTHTRY LHENILDYTE** |
|  |  | 6.7e+000 | 7.91e-13 | **LLQTTPDEID RAMYMCTGS EANDLAIRIA** |
|  |  |  | 1.09e-10 | **MGT RSTIMDTNS FRPEHAAELD** |
|  |  | 1.5e+001 | 2.11e-9 | **LDVYNNVASI GHCHPA VIEAVNEQMK** |
|  |  |  | 3.10e-8 | **EAYHGTSSLT SGCSPA LGTEQRLDDT** |
|  |  | 4.7e+002 | 3.29e-8 | **VGGLLLKELT KLMDKH EVVGDVRGAG** |
|  |  |  | 8.38e-8 | **HAAELDHATR KLTDKR NKILGESYRL** |
|  |  | 6.0e+002 | 6.19e-17 | **YRIQTDDLGE WFAQQIQQQIDDMN AKGIKFAGFL** |
|  |  |  | 3.55e-13 | **PVHLVKGLGQ YLWDAAGHQYLDVY NNVASIGHCH** |
|  |  | 1.2e+003 | 2.02e-7 | **SGGTGIIVSQ EAYHGT SSLTSGCSPA** |
|  |  |  | 2.64e-7 | **TNSFRPEHAA ELDHAT RKLTDKRNKI** |
|  |  | 7.1e+002 | 1.22e-7 | **AVDVVHANGG IFIADE VQPGFARTGD** |
|  |  |  | 1.22e-7 | **CTGSEANDLA IRIARE YSGGTGIIVS** |
|  |  | 1.5e+003 | 2.78e-11 | **NVLKLRPPLA FQTKDIDW LVGALDQALT** |
|  |  |  | 4.32e-8 | **SDGVMPNPVG FLQKAVDV VHANGGIFIA** |
|  |  | 2.0e+003 | 9.14e-10 | **LTSGCSPALG TEQRLDDT TRLVPAPDYY** |
|  |  |  | 9.75e-10 | **KMLNTHTRYL HENILDYT EELLQTTPDE** |
| 5 | [*Acinetobacter calcoaceticus*](https://www.uniprot.org/taxonomy/471)  III | 2.9e+000 | 1.47e-9 | **QSTPRGVGVM CQWYVE RAENATLWDQ** |
|  |  |  | 4.95e-8 | **QWYVERAENA TLWDQE GREFIDFAGG** |
|  |  | 3.4e+001 | 3.10e-21 | **TMAMTGKTAP YKRDFGVMPGGVFHARYP VAINNVSVDD** |
|  |  |  | 1.15e-19 | **ENAIKIARAA TKRHGVITFSGAFHGRSF MTMAMTGKTA** |
|  |  | 4.8e+001 | 1.27e-8 | **GGIAVLNTGH CHPKIM AAVTEQLGKF** |
|  |  |  | 6.22e-8 | **AMSKNLILLT CGKKAN VIRFLYPLTI** |
|  |  | 1.1e+002 | 2.79e-21 | **VLEPVQGEGG FNVAPQALMIRLRQICDEH GILLIADEVQ** |
|  |  |  | 5.00e-20 | **EQLGKFTHTA YQVVPYESYISLVERLIER APIAGPAKAA** |
|  |  | 1.9e+002 | 1.72e-8 | **DYHPEAKPDL MTMAKS LGGGFPISAV** |
|  |  |  | 6.89e-8 | **FSGAFHGRSF MTMAMT GKTAPYKRDF** |
|  |  | 2.0e+002 | 4.22e-8 | **TGFARTGKLF AMDYHP EAKPDLMTMA** |
|  |  |  | 8.38e-8 | **GAELVNVLED LKDSHS FVRDIRGIGS** |
|  |  | 2.3e+002 | 2.50e-19 | **MDNQYSALNARKQQS TPRGVGVMCQ** |
|  |  |  | 3.46e-15 | **VIRFLYPLTI PDAQFQAALAILKQS FDATSALKNM** |
|  |  | 1.3e+003 | 7.74e-9 | **VLDVIEEEKL CERANK LGAELVNVLE** |
|  |  |  | 7.47e-7 | **AALFTTGAEA VENAIK IARAATKRHG** |
|  |  | 1.5e+003 | 3.00e-8 | **KDSHSFVRDI RGIGSM IAVELDTAER** |
|  |  |  | 1.86e-7 | **LNARKQQSTP RGVGVM CQWYVERAEN** |
|  |  | 2.2e+003 | 2.25e-8 | **TCGKKANVIR FLYPLT IPDAQFQAAL** |
|  |  |  | 1.79e-7 | **KAIQDDAMSK NLILLT CGKKANVIRF** |
| 6 | [*Acinetobacter calcoaceticus*](https://www.uniprot.org/taxonomy/471)  IV | 7.4e+000 | 2.06e-11 | **TDKRAQLLGE SYRLFYRNPV HLVKGQGQYL** |
|  |  |  | 4.29e-11 | **VQPGFARTGD SFWGFGRHGV VPDIITTGKP** |
|  |  |  | 5.90e-11 | **VLAAFSDHIP YFNTFGGNPV AMAAAQAVLK** |
|  |  | 4.7e+000 | 2.79e-14 | **LLATAPAEID RAMYMCTGSE ANDLAIRIAR** |
|  |  |  | 1.04e-11 | **MVT RSTIMDTNSF REEHAAALDA** |
|  |  | 5.5e+001 | 1.15e-22 | **DVYNNVASIG HCHPAVIEAVNAQMQMLNTH TRYLHENILD** |
|  |  |  | 5.44e-21 | **IGFELVTDRD TKQPDKALALNLIEELRNTH RVLTSVAGPY** |
|  |  | 2.7e+002 | 7.52e-12 | **AVDVVHANGG IFIADEVQP GFARTGDSFW** |
|  |  |  | 9.70e-11 | **IKFAGFLADS IFSSDGVMP NPVGYLQKAV** |
|  |  | 6.9e+002 | 1.91e-8 | **VAMAAAQAVL KVIQEE QLQAHSKKVG** |
|  |  |  | 8.60e-8 | **ATDDLGEWFA KQIQQQ IDDMNAKGIK** |
|  |  | 5.0e+002 | 2.26e-13 | **TTRLVPAPDQ YRVATDDLGEW FAKQIQQQID** |
|  |  |  | 1.64e-11 | **GTGIIVSQEA YHGTSDLTSGC SPALGSGQAL** |
|  |  | 7.6e+002 | 5.97e-8 | **HAAALDADTR KLTDKR AQLLGESYRL** |
|  |  |  | 6.91e-8 | **VGAQLQAELS KLMDRH PRIGDVRGAG** |
|  |  | 5.7e+002 | 2.72e-10 | **HLVKGQGQYL WDAAGNKY LDVYNNVASI** |
|  |  |  | 1.17e-9 | **AKQIQQQIDD MNAKGIKF AGFLADSIFS** |
|  |  | 1.3e+003 | 1.43e-8 | **GVVPDIITTG KPMGNG IPVSGLLAKS** |
|  |  |  | 1.86e-7 | **NTHRVLTSVA GPYGNV LKLRPPLAFQ** |
|  |  | 1.3e+003 | 5.20e-8 | **AQMQMLNTHT RYLHEN ILDYSEALLA** |
|  |  |  | 2.78e-7 | **RSTIMDTNSF REEHAA ALDADTRKLT** |
| 7 | [*Acinetobacter calcoaceticus*](https://www.uniprot.org/taxonomy/471)  V | 1.6e+000 | 1.35e-8 | **KQQATPRGVG VMCQWY AEKAENATLW** |
|  |  |  | 1.81e-7 | **NYAMENGLLL LTCGKY GNVIRFLYPL** |
|  |  |  | 2.47e-7 | **VPAEFLKRLR AICDKH GILLVADEVQ** |
|  |  | 3.0e+001 | 1.12e-9 | **AAAHAVIDAI EEENLCD RANELGAELV** |
|  |  |  | 5.46e-9 | **VMCQWYAEKA ENATLWD KEGNQFIDFA** |
|  |  | 5.3e+001 | 1.40e-8 | **RHGIITFGNG FHGRSF MTMAMTGKTA** |
|  |  |  | 5.77e-8 | **RDFGVMPAGV FHARYP VPAKGISVDA** |
|  |  | 1.1e+002 | 2.71e-8 | **EQLTKFTHTA YQVTPY ESYVALAERI** |
|  |  |  | 7.29e-8 | **NQHSALNARK QQATPR GVGVMCQWYA** |
|  |  | 2.7e+002 | 1.69e-8 | **AVENAVKIAR CYTGRH GIITFGNGFH** |
|  |  |  | 2.62e-7 | **FIDFAGGIAV LNTGHR HPKVIAAVTE** |
|  |  | 4.6e+002 | 3.89e-8 | **MNHYETKADL ITMAKS LGGGFPISGV** |
|  |  |  | 7.84e-8 | **FGNGFHGRSF MTMAMT GKTAPYKRDF** |
|  |  | 5.8e+002 | 9.45e-10 | **ANELGAELVV SLKDIQQ ATGNVVTDIR** |
|  |  |  | 6.03e-8 | **KQGFAALKAG SAKAMEQ SA** |
|  |  | 6.7e+002 | 4.17e-9 | **MTMAMTGKTA PYKRDF GVMPAGVFHA** |
|  |  |  | 1.15e-6 | **RINERAPIAG PAKSAF FTTGAEAVEN** |
|  |  | 8.2e+002 | 1.72e-8 | **EGGFHVVPAE FLKRLR AICDKHGILL** |
|  |  |  | 1.28e-7 | **TCGKYGNVIR FLYPLT IPAEQFRQGL** |
|  |  | 1.2e+003 | 2.70e-8 | **ETAEQAKVVQ NYAMEN GLLLLTCGKY** |
|  |  |  | 2.51e-7 | **VQSGFARTGK LFAMNH YETKADLITM** |
| 8 | [*Acinetobacter calcoaceticus*](https://www.uniprot.org/taxonomy/471)  VI | 7.4e+000 | 2.06e-11 | **TDKRAQLLGE SYRLFYRNPV HLVKGQGQYL** |
|  |  |  | 4.29e-11 | **VQPGFARTGD SFWGFGRHGV VPDIITTGKP** |
|  |  |  | 5.90e-11 | **VLAAFSDHIP YFNTFGGNPV AMAAAQAVLK** |
|  |  | 4.7e+000 | 2.79e-14 | **LLATAPAEID RAMYMCTGSE ANDLAIRIAR** |
|  |  |  | 1.04e-11 | **MVT RSTIMDTNSF REEHAAALDA** |
|  |  | 5.5e+001 | 1.15e-22 | **DVYNNVASIG HCHPAVIEAVNAQMQMLNTH TRYLHENILD** |
|  |  |  | 5.44e-21 | **IGFELVTDRD TKQPDKALALNLIEELRNTH RVLTSVAGPY** |
|  |  | 2.7e+002 | 7.52e-12 | **AVDVVHANGG IFIADEVQP GFARTGDSFW** |
|  |  |  | 9.70e-11 | **IKFAGFLADS IFSSDGVMP NPVGYLQKAV** |
|  |  | 6.9e+002 | 1.91e-8 | **VAMAAAQAVL KVIQEE QLQAHSKKVG** |
|  |  |  | 8.60e-8 | **ATDDLGEWFA KQIQQQ IDDMNAKGIK** |
|  |  | 5.0e+002 | 2.26e-13 | **TTRLVPAPDQ YRVATDDLGEW FAKQIQQQID** |
|  |  |  | 1.64e-11 | **GTGIIVSQEA YHGTSDLTSGC SPALGSGQAL** |
|  |  | 7.6e+002 | 5.97e-8 | **HAAALDADTR KLTDKR AQLLGESYRL** |
|  |  |  | 6.91e-8 | **VGAQLQAELS KLMDRH PRIGDVRGAG** |
|  |  | 5.7e+002 | 2.72e-10 | **HLVKGQGQYL WDAAGNKY LDVYNNVASI** |
|  |  |  | 1.17e-9 | **AKQIQQQIDD MNAKGIKF AGFLADSIFS** |
|  |  | 1.3e+003 | 1.43e-8 | **GVVPDIITTG KPMGNG IPVSGLLAKS** |
|  |  |  | 1.86e-7 | **NTHRVLTSVA GPYGNV LKLRPPLAFQ** |
|  |  | 1.3e+003 | 5.20e-8 | **AQMQMLNTHT RYLHEN ILDYSEALLA** |
|  |  |  | 2.78e-7 | **RSTIMDTNSF REEHAA ALDADTRKLT** |
| 9 | [*Clostridium* sp](https://www.uniprot.org/taxonomy/59620). I | 1.3e-001 | 1.21e-14 | **EAVKKQSERY FHGMFNIVTH EGYVALAEKL** |
|  |  |  | 1.12e-12 | **AFTKRPNIIV FSGAFHGRTM LTMAMTSKKA** |
|  |  | 1.1e+001 | 1.52e-17 | **LLVADEVQSG FCRTGRMFASDYW KDADAQPDIV** |
|  |  |  | 2.87e-14 | **AVPCGVIGGT FCGNPLACASALQ VIEIMEKENF** |
|  |  | 5.9e+001 | 7.61e-10 | **EGAMLEDVDG NYFLDW VGGVGVLNIG** |
|  |  |  | 4.67e-7 | **IAPVKGTKRK TYFANS GAEADENAVK** |
|  |  | 1.6e+002 | 1.52e-8 | **PNPTLVSEVV QECIQN GLIIENAGIY** |
|  |  |  | 5.76e-8 | **AMIGLEIVKD QESKQP NPTLVSEVVQ** |
|  |  | 6.9e+002 | 3.17e-9 | **KKAYAYGMGP FPDGVYR AEFPYLYRKL** |
|  |  |  | 1.27e-8 | **ASDYWKDADA QPDIVTT AKSIAGGIPL** |
|  |  | 1.1e+003 | 4.48e-10 | **VPAPIEWVKA VRKICDKY GILLVADEVQ** |
|  |  |  | 1.22e-9 | **EARAVEIEKK VMKRYQKM QKKLPVIGDV** |
|  |  | 1.6e+003 | 3.89e-10 | **VEPLQGEGGF VPAPIEWV KAVRKICDKY** |
|  |  |  | 6.00e-9 | **AKWIARRGEA VPSAIKCV YPIVIARGEG** |
|  |  | 1.6e+003 | 1.05e-7 | **IKCVYPIVIA RGEGAM LEDVDGNYFL** |
|  |  |  | 2.18e-7 | **QKKLPVIGDV RGIGAM IGLEIVKDQE** |
|  |  | 3.7e+003 | 4.43e-8 | **VGGVGVLNIG YSRPEL IEAVKKQSER** |
|  |  |  | 2.43e-7 | **ENAVKIAKAF TKRPNI IVFSGAFHGR** |
|  |  | 5.5e+003 | 6.14e-8 | **FSGAFHGRTM LTMAMT SKKAYAYGMG** |
|  |  |  | 2.07e-7 | **TTAKSIAGGI PLSAIT AREEIMEAVP** |
| 8 | [*Clostridium* sp](https://www.uniprot.org/taxonomy/59620). II | 5.4e+001 | 9.60e-9 | **ASGIATNALG HCHPKV IAAAEKQLHT** |
|  |  |  | 5.56e-8 | **MTQ HERSKV LLPPMAMHAE** |
|  |  | 1.2e+002 | 1.27e-8 | **RTPYKMEDGK CPKEYF TQFDEIFKKI** |
|  |  |  | 1.29e-7 | **AAYRKNYEPM MPSVYF AEYPYLFRTP** |
|  |  | 1.5e+002 | 3.65e-8 | **EVQTGFGRTG NLYAWQ TLGVEPDIFT** |
|  |  |  | 1.57e-7 | **VKKMGEVVRG KFYDLQ KKYDVIGDVR** |
|  |  | 1.8e+002 | 1.33e-11 | **RDLLLLNCGA DHNNIRLI PPLNVDEATL** |
|  |  |  | 7.92e-10 | **EWLKYVRELC DKHGIMLI FDEVQTGFGR** |
|  |  | 2.8e+002 | 2.45e-8 | **GKCPKEYFTQ FDEIFK KIVDPSMVAA** |
|  |  |  | 8.89e-8 | **LCDKHGIMLI FDEVQT GFGRTGNLYA** |
|  |  | 2.8e+002 | 1.15e-8 | **VDEATLDTVF QIIDES IAAALKG** |
|  |  |  | 9.10e-8 | **YFTQFDEIFK KIVDPS MVAAIMMEPV** |
|  |  | 5.5e+002 | 1.14e-9 | **AMHAETTPCV VRGEGCY LYTEDGRKIL** |
|  |  |  | 1.45e-8 | **SMVAAIMMEP VQGEGGY VVPPVEWLKY** |
|  |  | 2.1e+002 | 2.29e-8 | **EGGYVVPPVE WLKYVR ELCDKHGIML** |
|  |  |  | 1.45e-7 | **VFGKKEIMDK WAKGAH GGTYGGNPVS** |
|  |  | 8.3e+002 | 5.34e-8 | **KPEDNAPDGA LCAAVQ AEALKRDLLL** |
|  |  |  | 9.27e-8 | **HGGTYGGNPV SCAASL AVLEELYEGG** |
|  |  | 8.8e+002 | 1.28e-8 | **QRPVIISMKN AFHGRT MATATITTSN** |
|  |  |  | 3.75e-7 | **ERSKVLLPPM AMHAET TPCVVRGEGC** |
| 9 | *Salmonella typhimurium* | 1.4e+001 | 2.13e-10 | **IHPIFAERAE NCRVWD VEGREYLDFA** |
|  |  |  | 2.71e-7 | **MPGHVYRALY PCPLHN ISDDDAIASI** |
|  |  | 3.6e+001 | 2.48e-12 | **VLAYEPYLAL CERMNQKVP GDFAKKTLLV** |
|  |  |  | 2.04e-10 | **MNTNNAL MQRRHNAVP RGVGQIHPIF** |
|  |  | 6.8e+001 | 1.23e-8 | **HGRTHYTLSL TGKVHP YSAGMGLMPG** |
|  |  |  | 7.73e-8 | **DFAGGIAVLN TGHLHP GIVSAVEAQL** |
|  |  | 9.3e+001 | 1.92e-10 | **PAFMQRLRAL CDQHGIM LIADEVQSGA** |
|  |  |  | 2.38e-8 | **EAQLKKLSHT CFQVLAY EPYLALCERM** |
|  |  | 2.2e+002 | 2.98e-9 | **VQGEGGFYAA SPAFMQR LRALCDQHGI** |
|  |  |  | 6.06e-9 | **NAVPRGVGQI HPIFAER AENCRVWDVE** |
|  |  | 1.1e+002 | 6.59e-9 | **RAREKGLILL SCGPYY NILRILVPLT** |
|  |  |  | 1.08e-7 | **VHPYSAGMGL MPGHVY RALYPCPLHN** |
|  |  | 5.7e+002 | 3.24e-8 | **CERMNQKVPG DFAKKT LLVTTGSEAV** |
|  |  |  | 1.85e-7 | **EQMGVAADIT TFAKSI AGGFPLAGVT** |
|  |  | 8.3e+002 | 3.24e-8 | **CAAALAVLDI FEQENL LQKANTLGKT** |
|  |  |  | 1.55e-7 | **QSGAGRTGTL FAMEQM GVAADITTFA** |
|  |  | 1.1e+003 | 2.63e-18 | **RILVPLTIEA SQIRQGLEIIAQCFDE AKQA** |
|  |  |  | 1.66e-16 | **NLLQKANTLG KTLRDGLMEIAETHRE IGDVRGLGAM** |
|  |  | 1.2e+003 | 6.72e-8 | **DVRGLGAMIA IELFEN GDPGKPNAAL** |
|  |  |  | 3.16e-7 | **AKKTLLVTTG SEAVEN AVKIARAATK** |

**
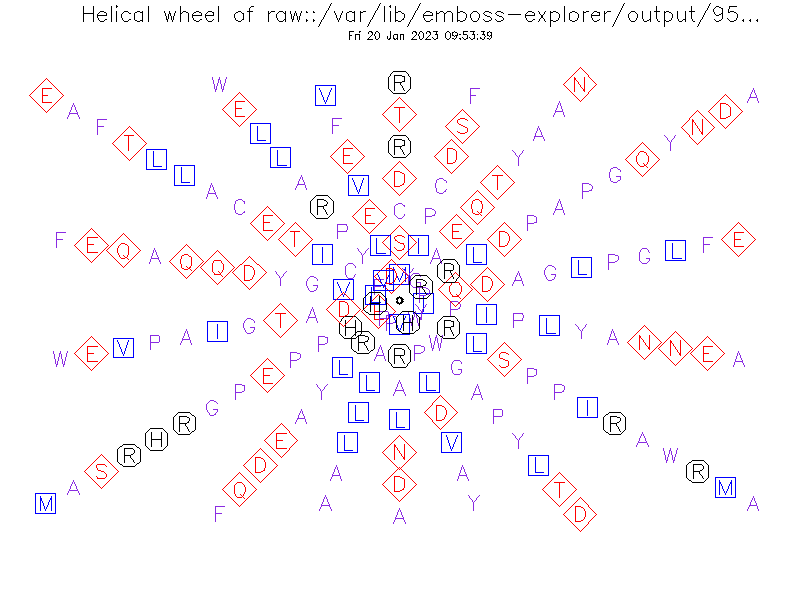
**

(a)

**
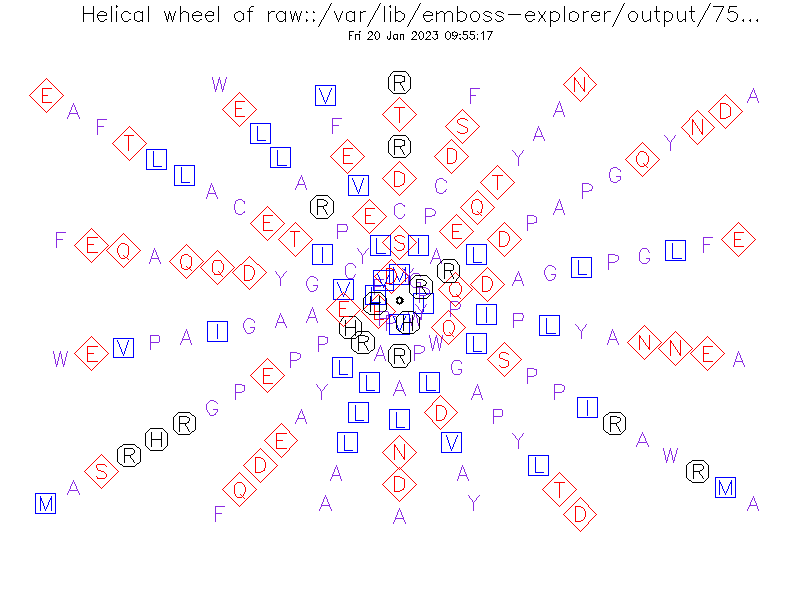
**

(b)

**
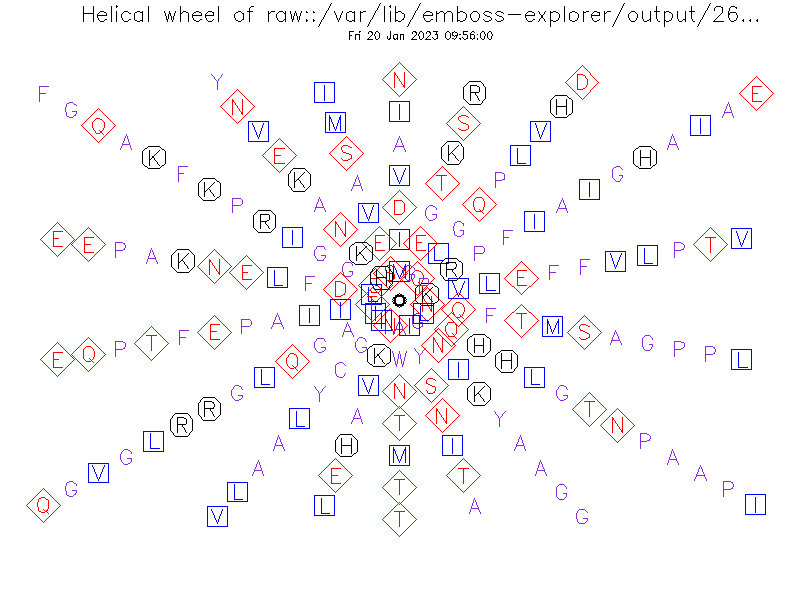
**

(c)

**
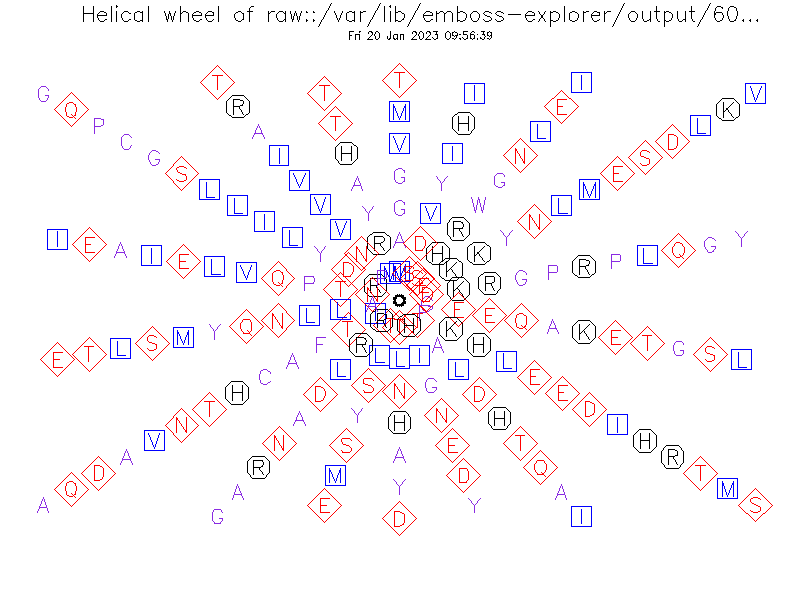
**

(d)

**
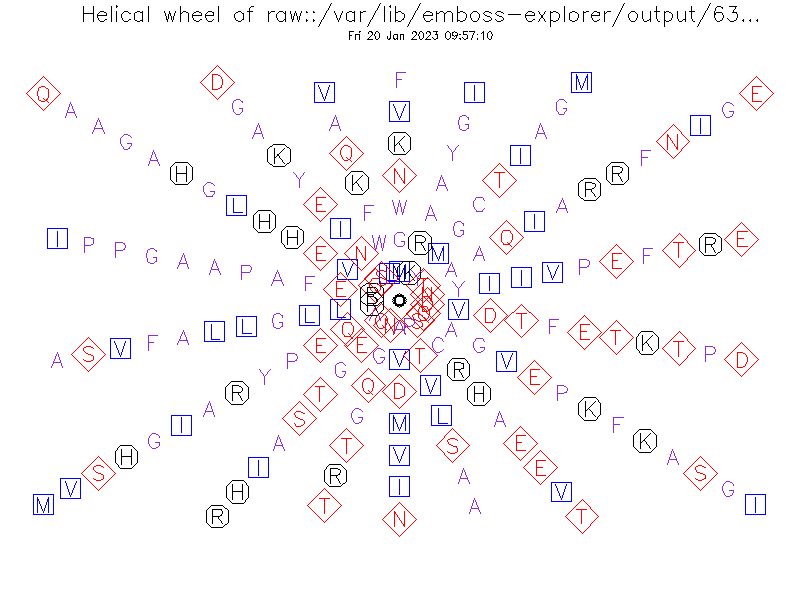
**

(e)


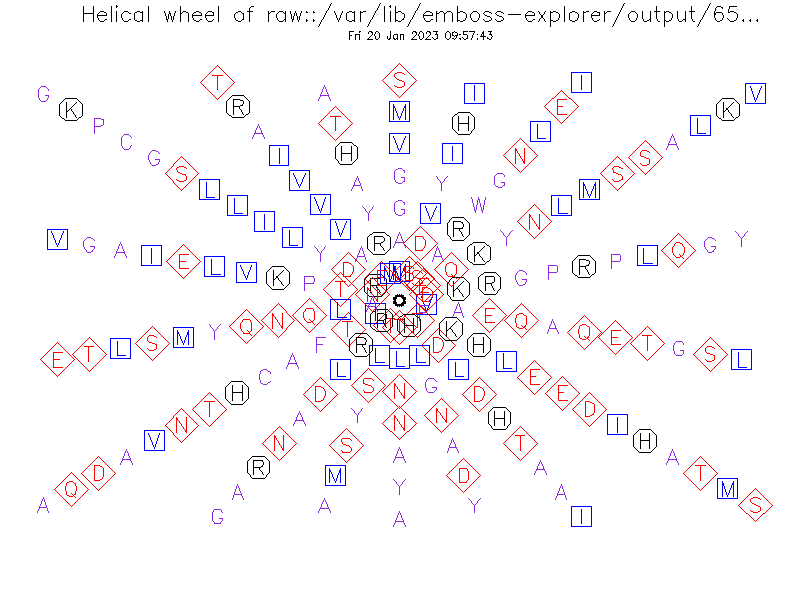


(f)


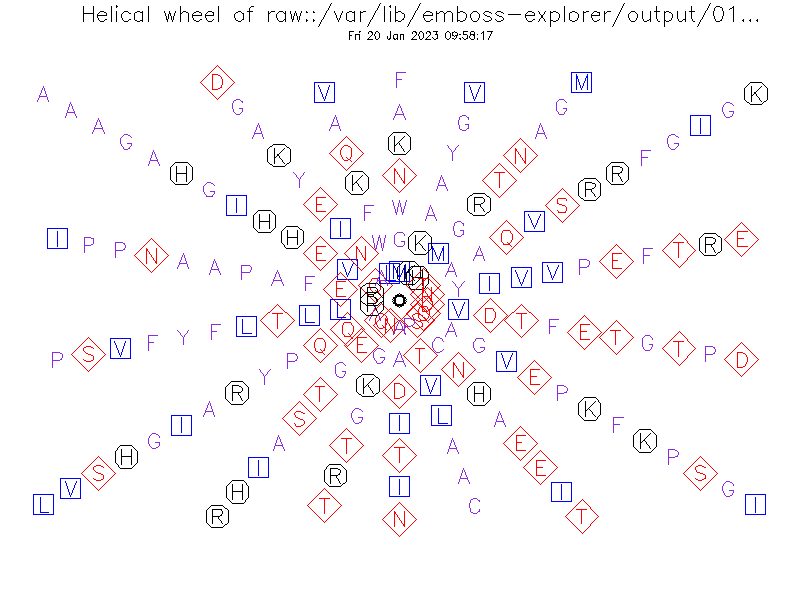


(g)


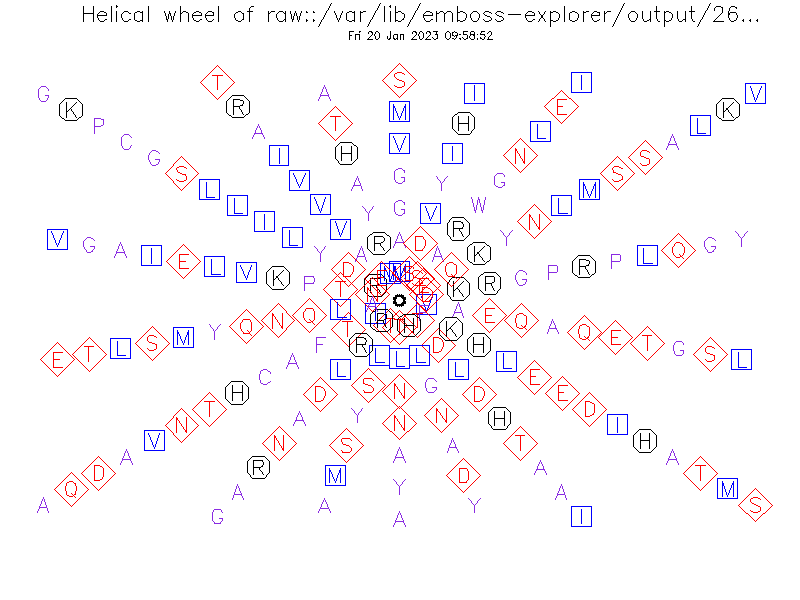


(h)


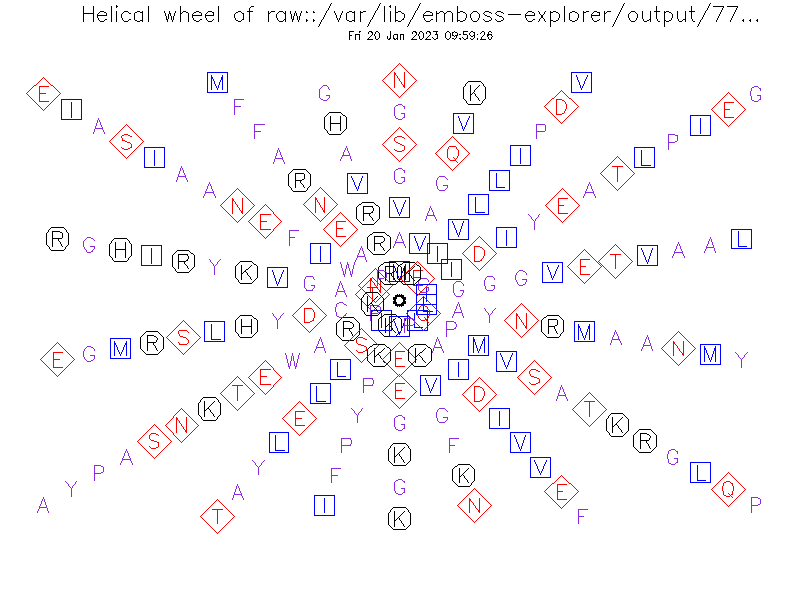


(i)


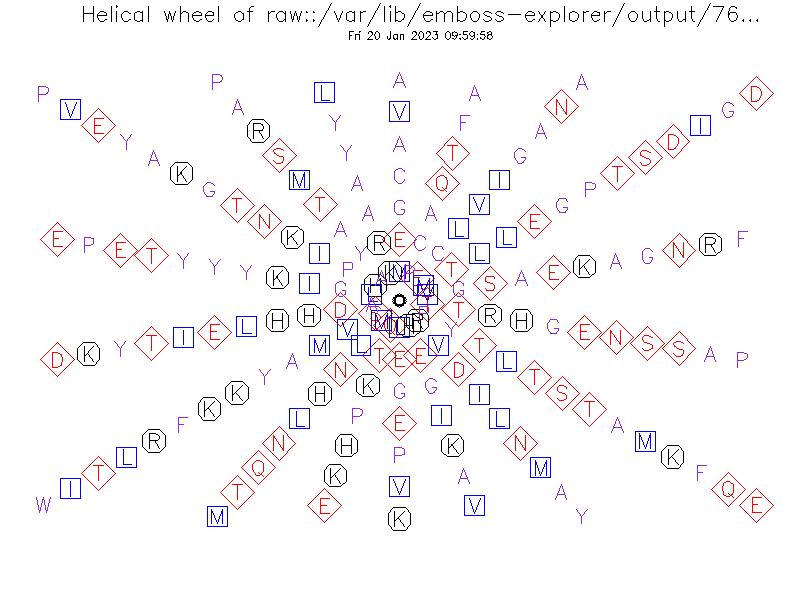


(j)


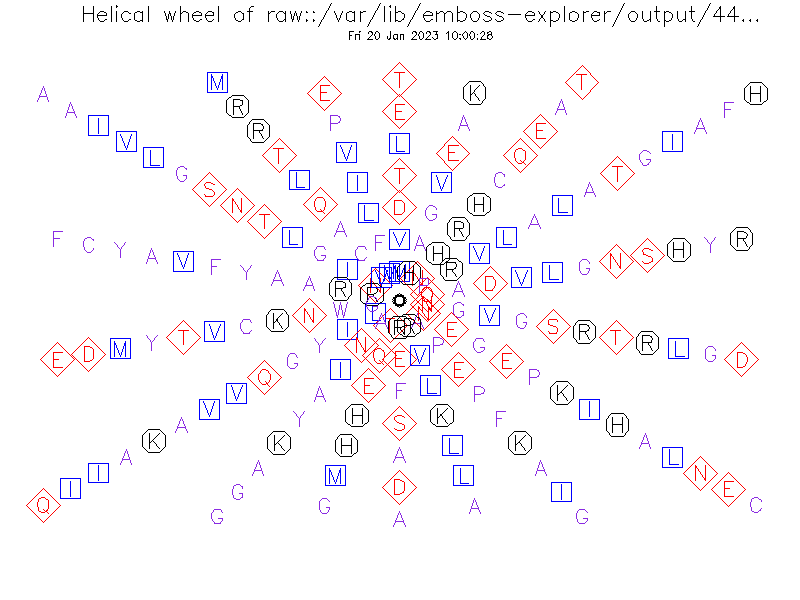


(k)

**Supplementary data Figure 1:** Helical wheel diagrams showing aliphatic, hydrophilic and positively charged residues in GABAT proteins of bacteria found in GIT of normal individuals and MS patients

Squares = aliphatic residues, Diamonds = hydrophilic residues, Octagons = positively charged residues. (a) [*Faecalibacterium* sp. An58](https://www.uniprot.org/taxonomy/1965648), (b) [*Faecalibacterium* sp. An121](https://www.uniprot.org/taxonomy/1965550), (c) [*Acinetobacter calcoaceticus*](https://www.uniprot.org/taxonomy/471) I, (d) [*Acinetobacter calcoaceticus*](https://www.uniprot.org/taxonomy/471) II, (e) [*Acinetobacter calcoaceticus*](https://www.uniprot.org/taxonomy/471) III, (f) [*Acinetobacter calcoaceticus*](https://www.uniprot.org/taxonomy/471) IV, (g) [*Acinetobacter calcoaceticus*](https://www.uniprot.org/taxonomy/471) V, (h) [*Acinetobacter calcoaceticus*](https://www.uniprot.org/taxonomy/471) VI, (i) [*Clostridium* sp](https://www.uniprot.org/taxonomy/59620). I, (j) [*Clostridium* sp](https://www.uniprot.org/taxonomy/59620). II, (k) *Salmonella typhimurium*


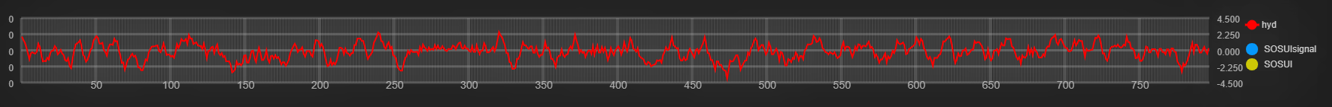


(a)


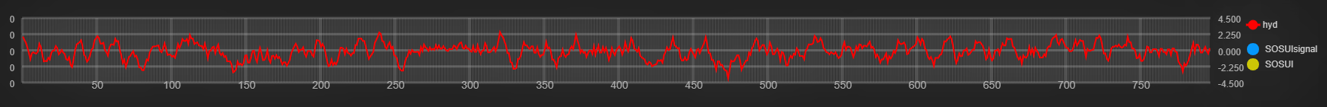


(b)


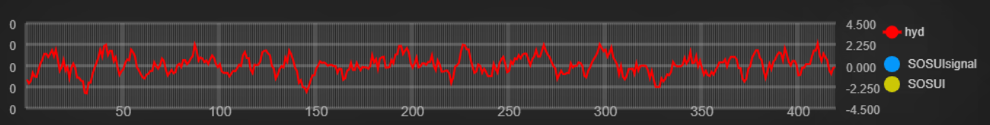


(c)


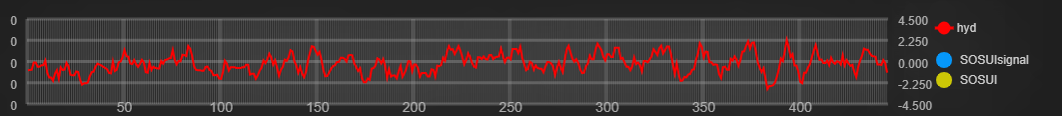


(d)


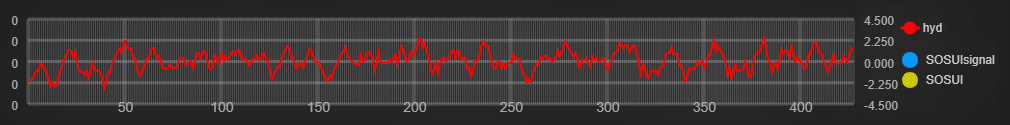


(e)


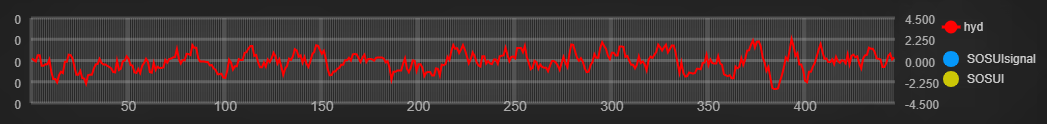


(f)


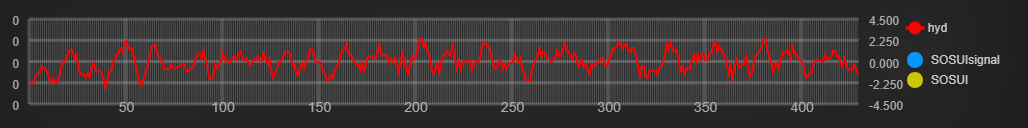


(g)


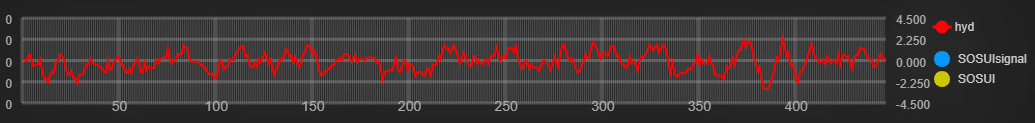


(h)


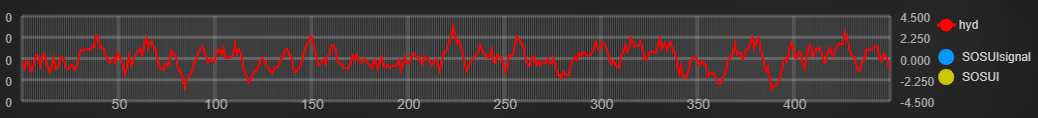


(i)


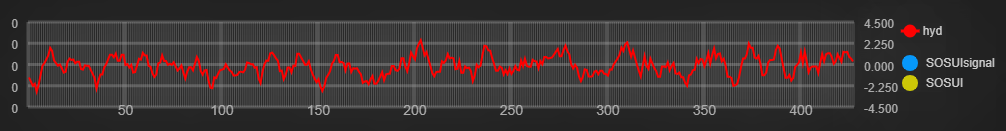


(j)


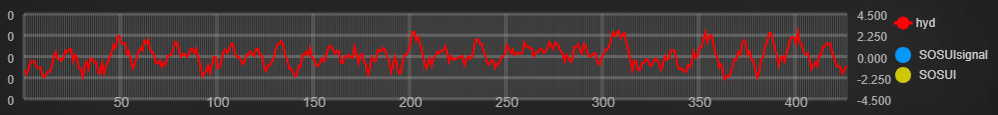


(k)

**Supplementary data Figure 2:** Hydropathy and charge plots predicted using SOSUI online tool in GABAT proteins of bacteria found in GIT of normal individuals and MS patients

(a) [*Faecalibacterium* sp. An58](https://www.uniprot.org/taxonomy/1965648), (b) [*Faecalibacterium* sp. An121](https://www.uniprot.org/taxonomy/1965550), (c) [*Acinetobacter calcoaceticus*](https://www.uniprot.org/taxonomy/471) I, (d) [*Acinetobacter calcoaceticus*](https://www.uniprot.org/taxonomy/471) II, (e) [*Acinetobacter calcoaceticus*](https://www.uniprot.org/taxonomy/471) III, (f) [*Acinetobacter calcoaceticus*](https://www.uniprot.org/taxonomy/471) IV, (g) [*Acinetobacter calcoaceticus*](https://www.uniprot.org/taxonomy/471) V, (h) [*Acinetobacter calcoaceticus*](https://www.uniprot.org/taxonomy/471) VI, (i) [*Clostridium* sp](https://www.uniprot.org/taxonomy/59620). I, (j) [*Clostridium* sp](https://www.uniprot.org/taxonomy/59620). II, (k) *Salmonella typhimurium*


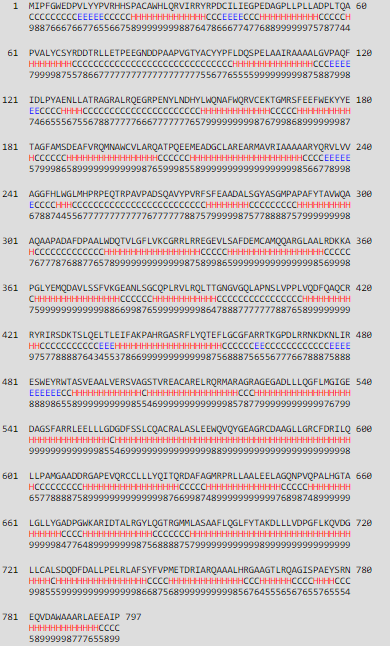

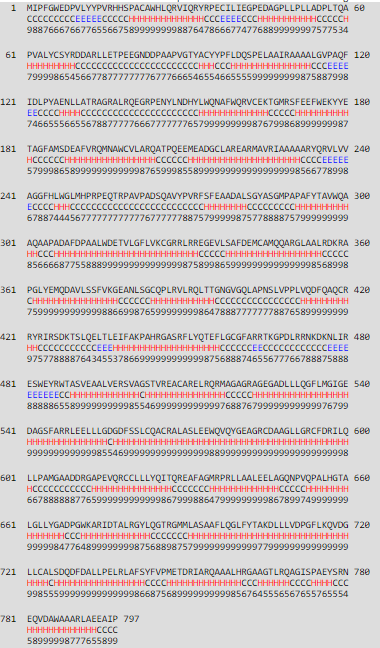


1. (b)


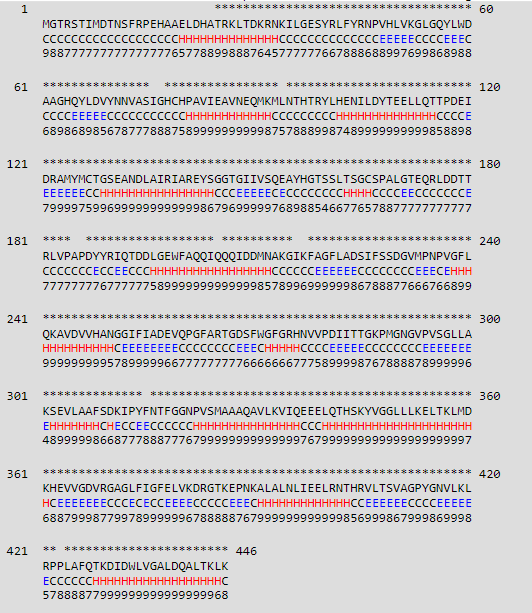

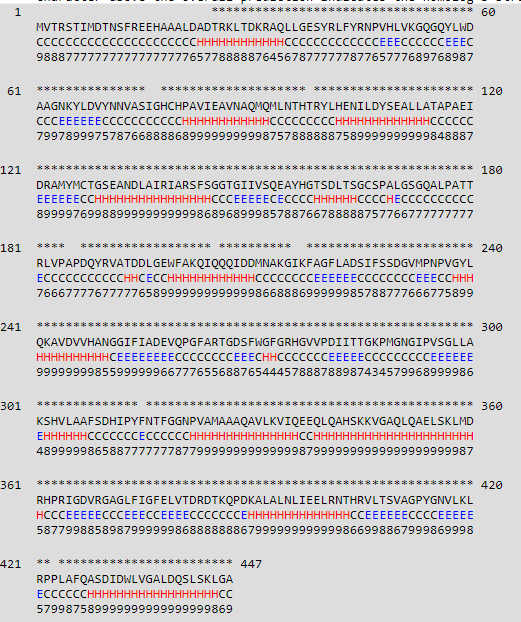


(c) (d)


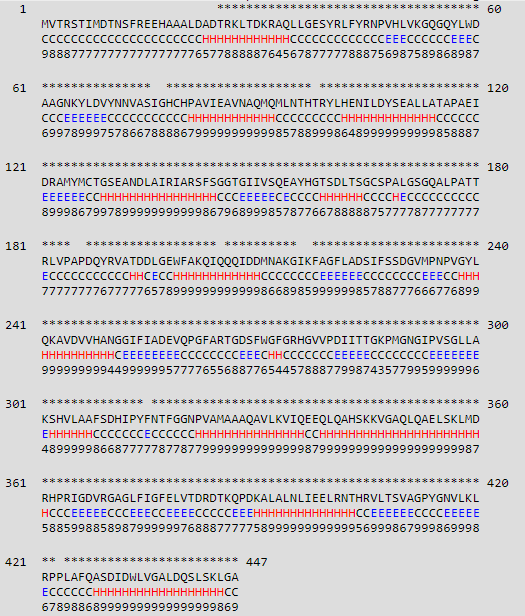

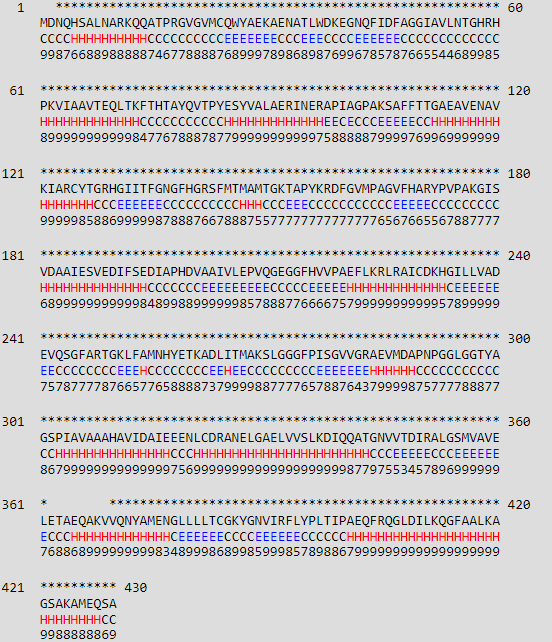


(e) (f)


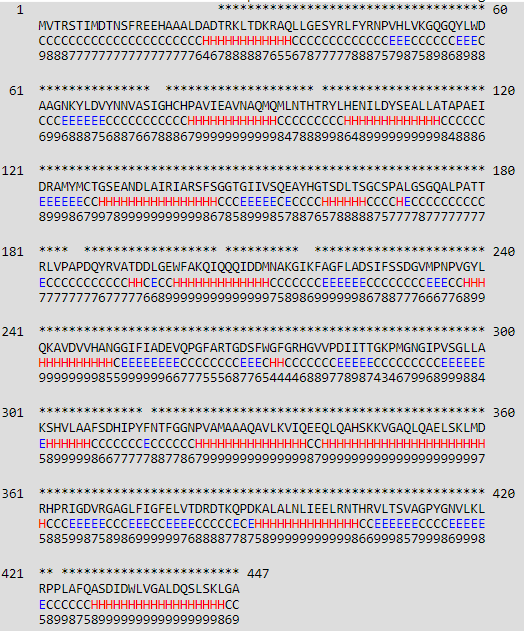

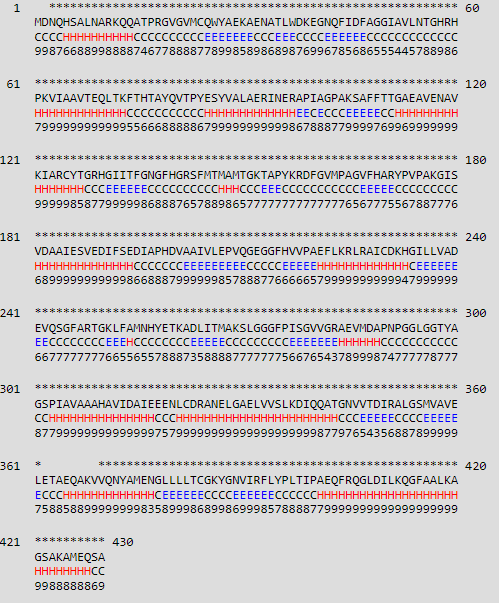

 (g) (h)


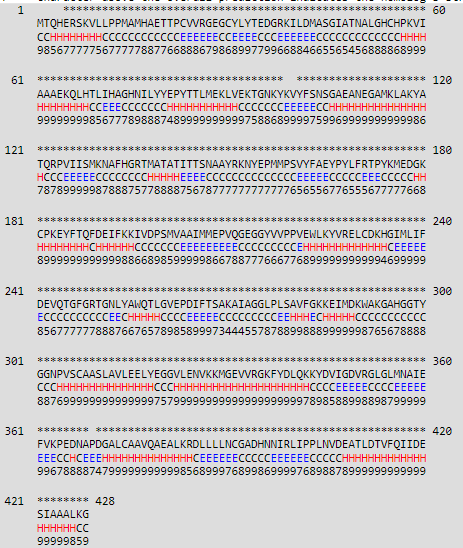

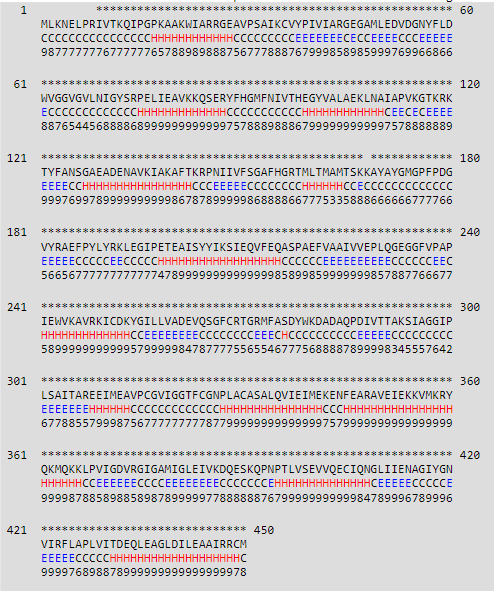

 (i) (j)


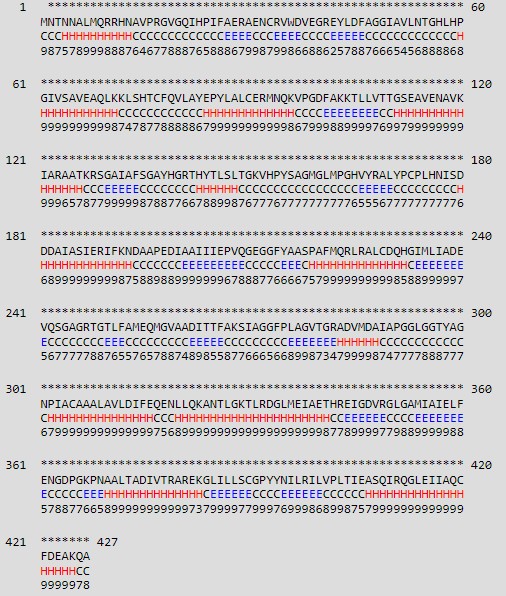


(k)

**Supplementary data Figure 3:** Prediction of secondary structures of GABAT proteins of bacteria found in GIT of normal individuals and MS patients

**H** = Helix, **E** = Beta Strand, **C** = Coil, **T** = Membrane helix, **B** = Membrane strand, **S** = Signal peptide, **c** = Cleavage site

(a) [*Faecalibacterium* sp. An58](https://www.uniprot.org/taxonomy/1965648), (b) [*Faecalibacterium* sp. An121](https://www.uniprot.org/taxonomy/1965550), (c) [*Acinetobacter calcoaceticus*](https://www.uniprot.org/taxonomy/471) I, (d) [*Acinetobacter calcoaceticus*](https://www.uniprot.org/taxonomy/471) II, (e) [*Acinetobacter calcoaceticus*](https://www.uniprot.org/taxonomy/471) III, (f) [*Acinetobacter calcoaceticus*](https://www.uniprot.org/taxonomy/471) IV, (g) [*Acinetobacter calcoaceticus*](https://www.uniprot.org/taxonomy/471) V, (h) [*Acinetobacter calcoaceticus*](https://www.uniprot.org/taxonomy/471) VI, (i) [*Clostridium* sp](https://www.uniprot.org/taxonomy/59620). I, (j) [*Clostridium* sp](https://www.uniprot.org/taxonomy/59620). II, (k) *Salmonella typhimurium*


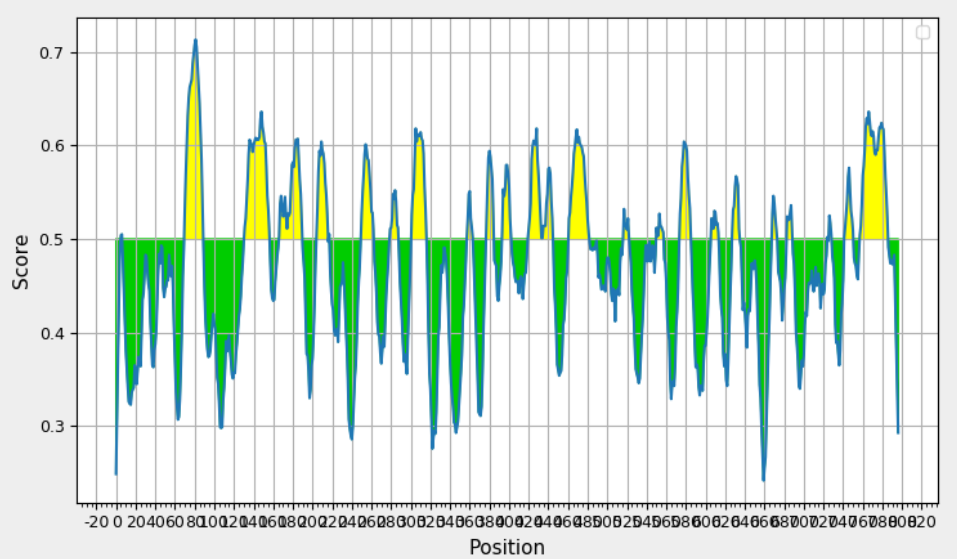


(a)


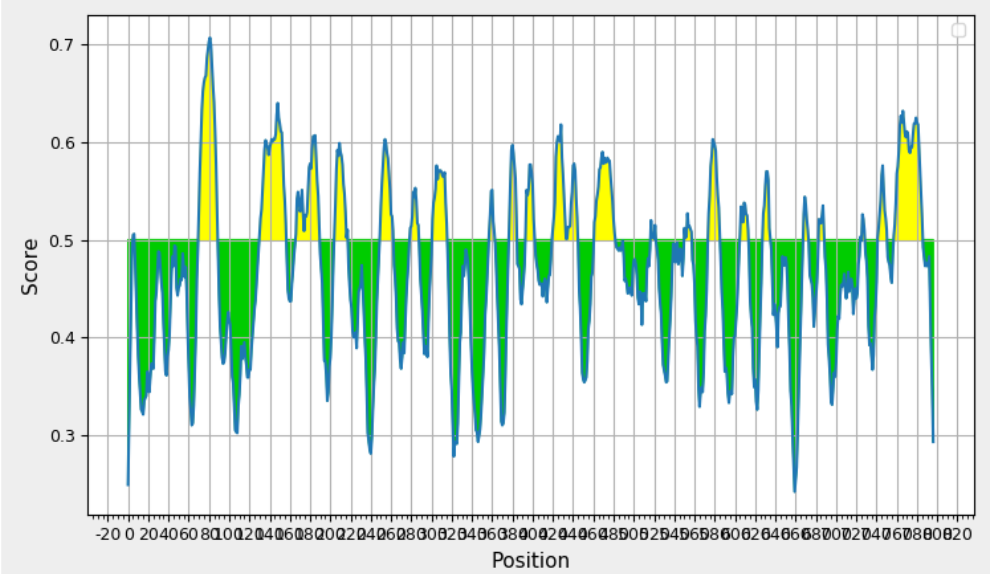


(b)


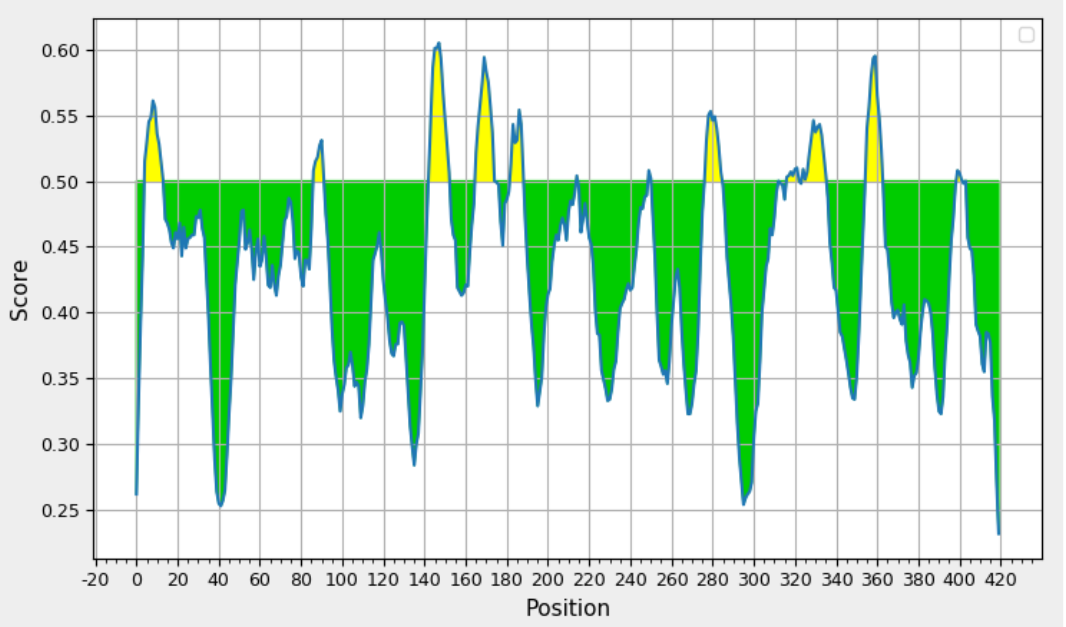


(c)


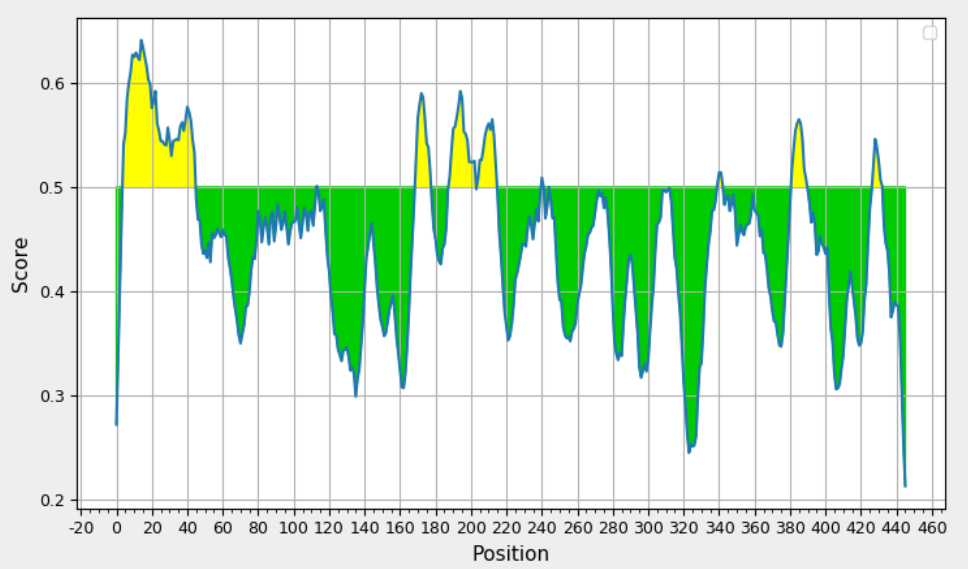


(d)


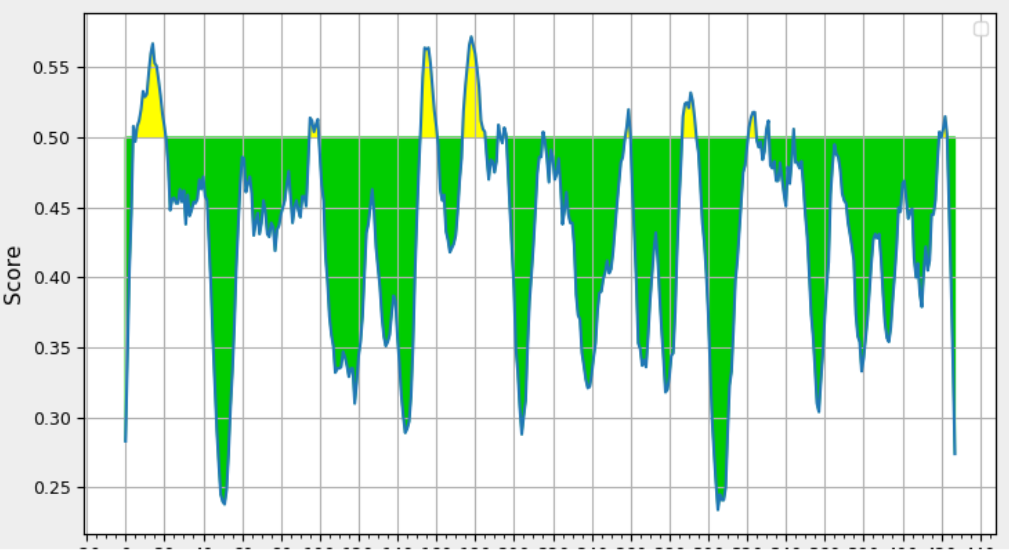


(e)


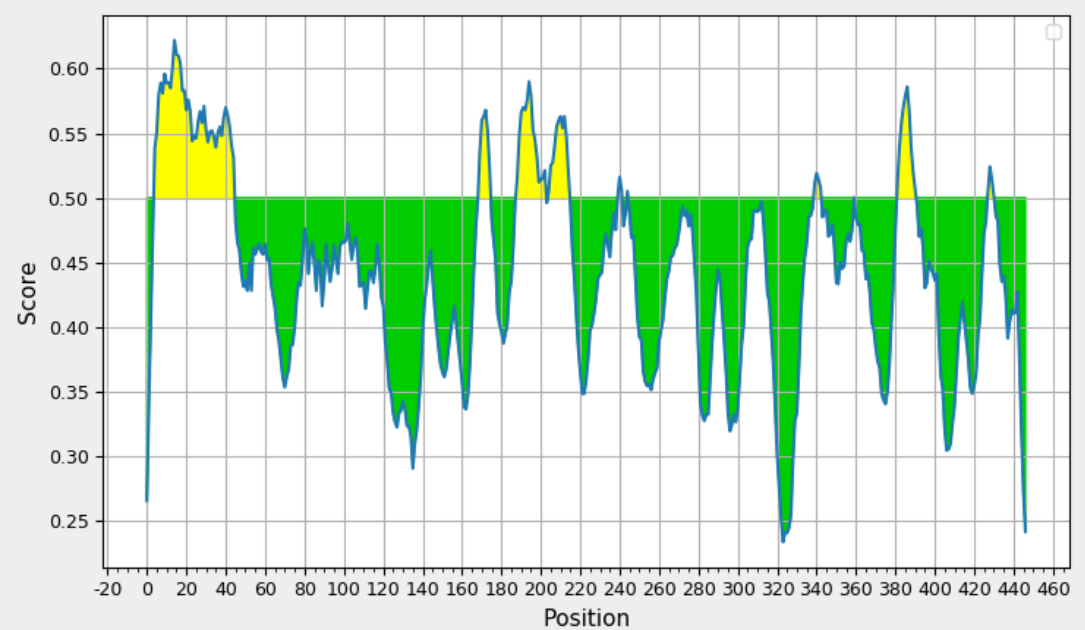


(f)


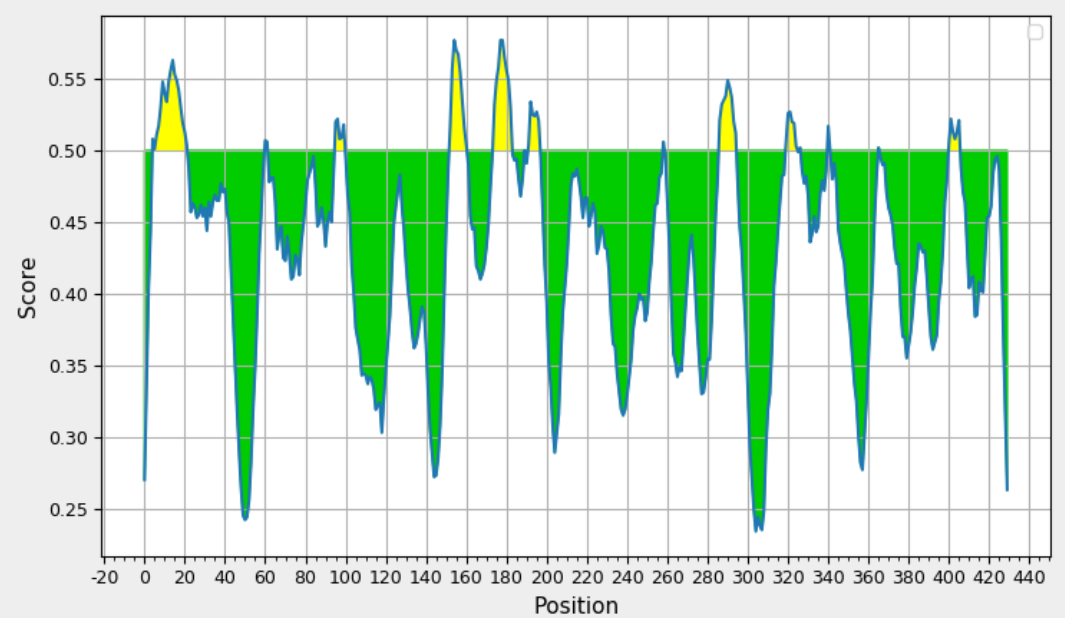


(g)


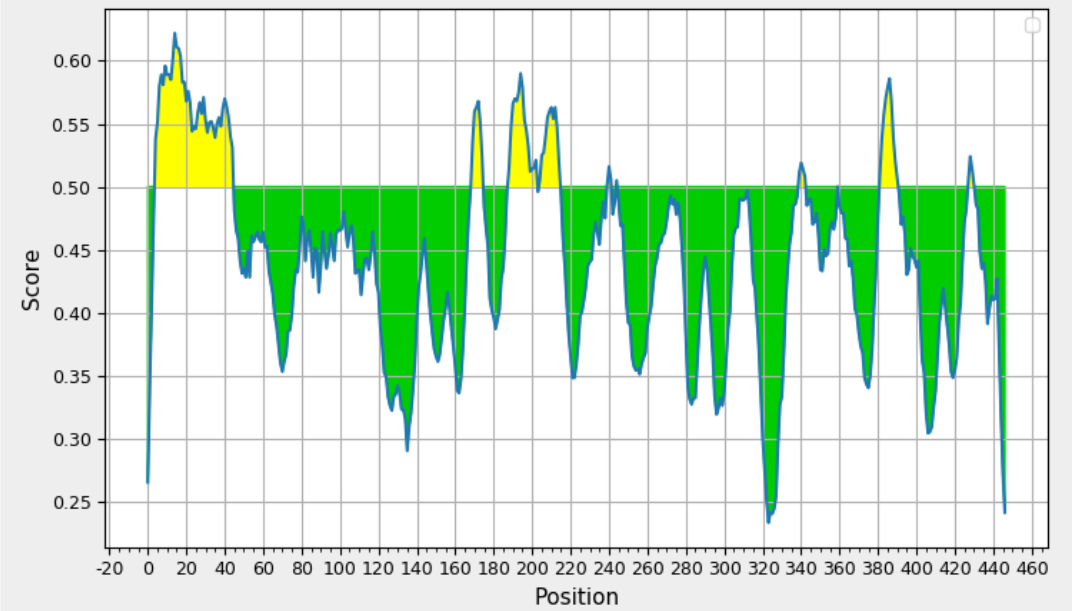


(h)


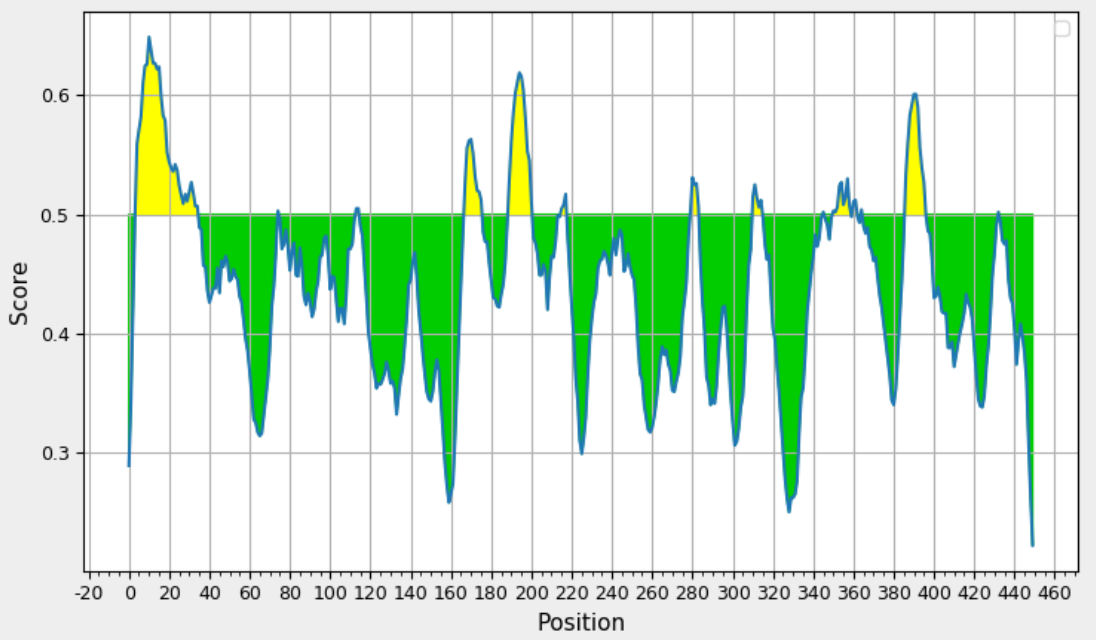


(i)


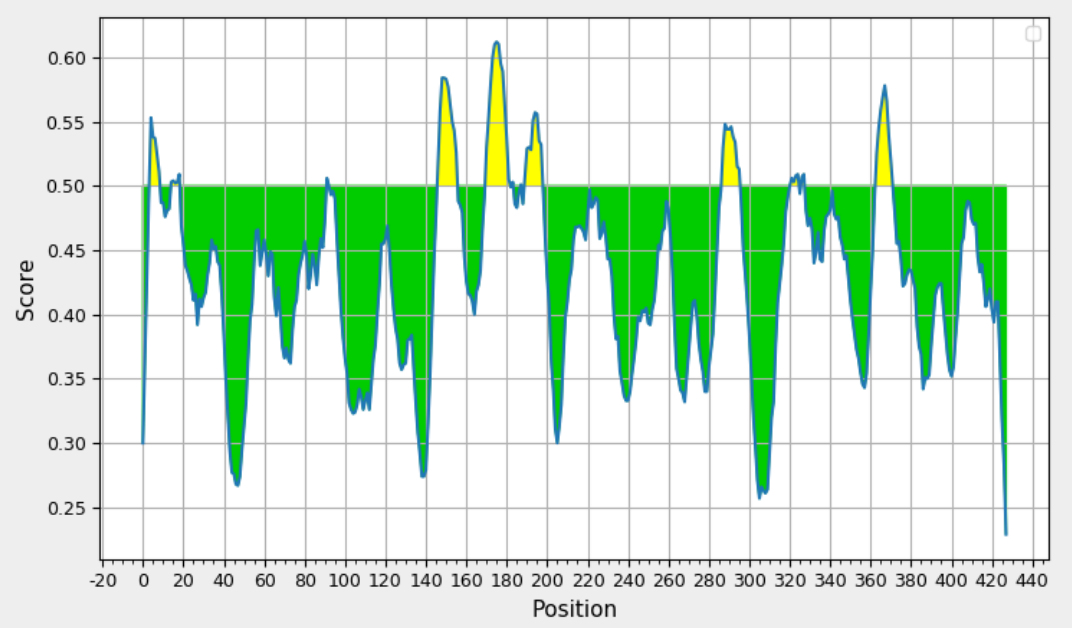


(j)


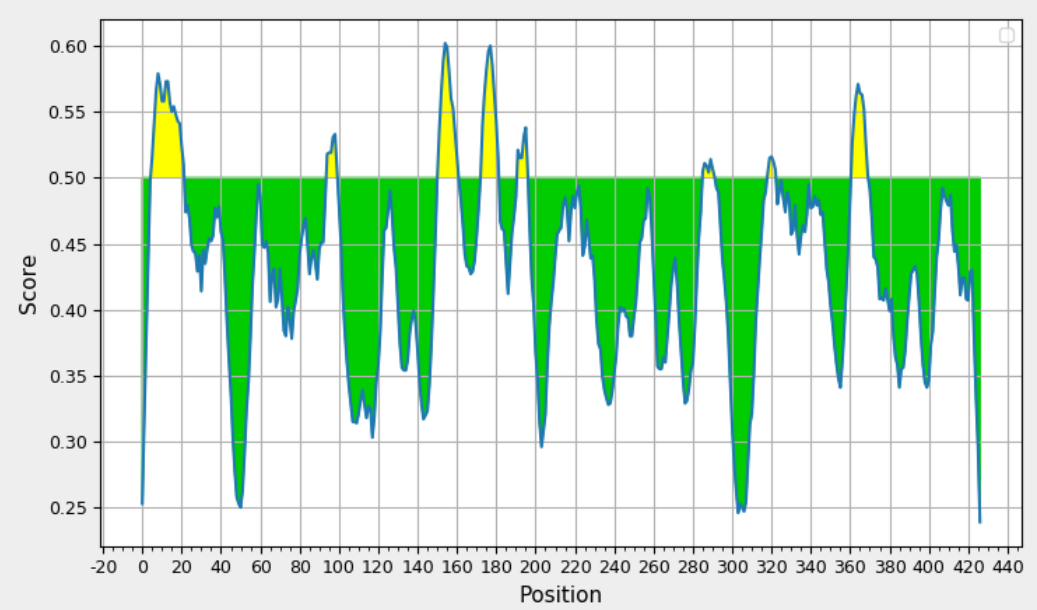


(k)

**Supplementary data Figure 4:** Graphical representation of scores of B-cell epitopes of GABAT proteins found in GIT bacteria of normal individuals and MS patients, predicted using IEDB

**H** = Helix, **E** = Beta Strand, **C** = Coil, **T** = Membrane helix, **B** = Membrane strand, **S** = Signal peptide, **c** = Cleavage site

(a) [*Faecalibacterium* sp. An58](https://www.uniprot.org/taxonomy/1965648), (b) [*Faecalibacterium* sp. An121](https://www.uniprot.org/taxonomy/1965550), (c) [*Acinetobacter calcoaceticus*](https://www.uniprot.org/taxonomy/471) I, (d) [*Acinetobacter calcoaceticus*](https://www.uniprot.org/taxonomy/471) II, (e) [*Acinetobacter calcoaceticus*](https://www.uniprot.org/taxonomy/471) III, (f) [*Acinetobacter calcoaceticus*](https://www.uniprot.org/taxonomy/471) IV, (g) [*Acinetobacter calcoaceticus*](https://www.uniprot.org/taxonomy/471) V, (h) [*Acinetobacter calcoaceticus*](https://www.uniprot.org/taxonomy/471) VI, (i) [*Clostridium* sp](https://www.uniprot.org/taxonomy/59620). I, (j) [*Clostridium* sp](https://www.uniprot.org/taxonomy/59620). II, (k) *Salmonella typhimurium*


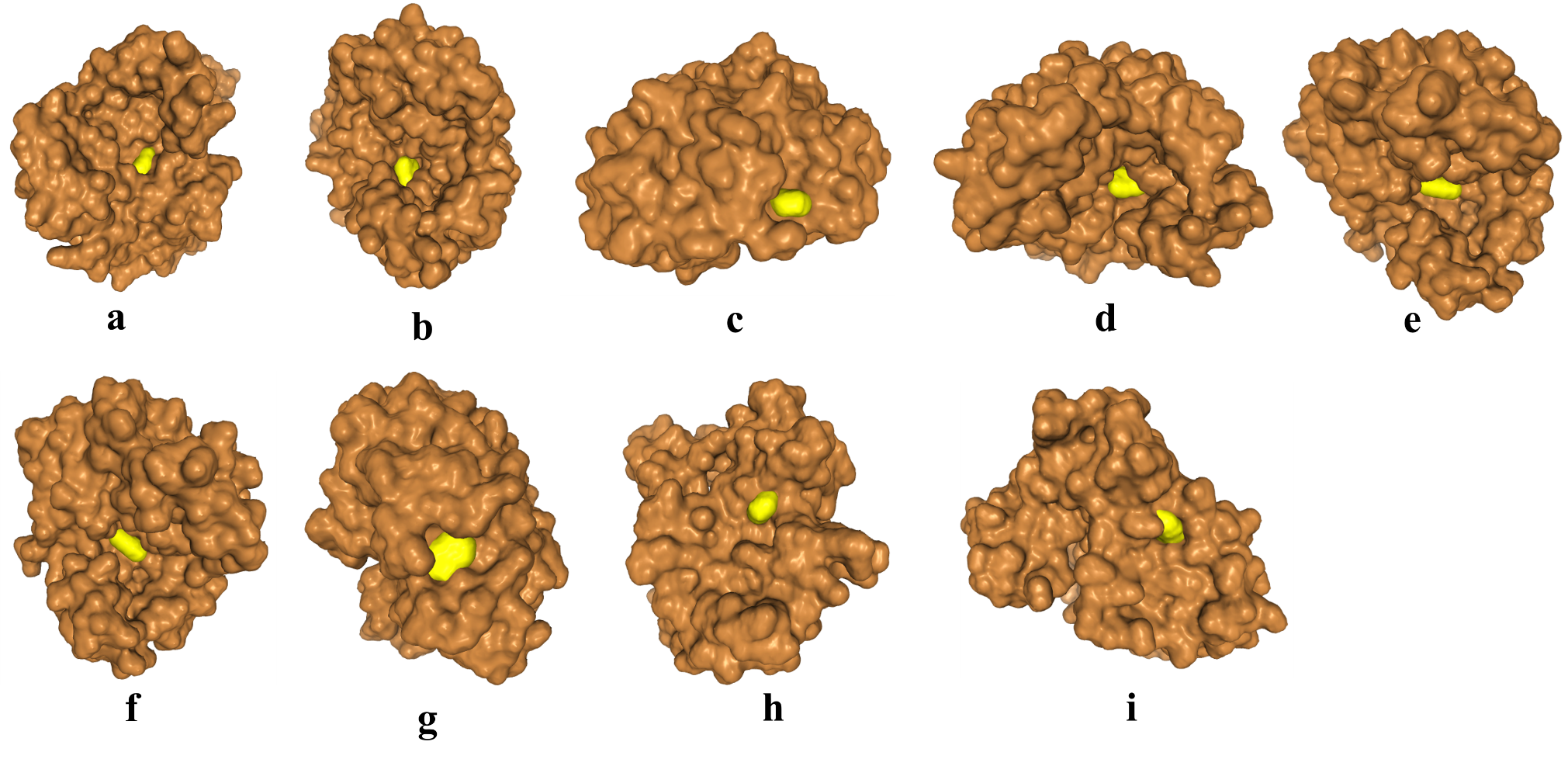


**Supplementary data Figure S5:** Docking analysis of GABAT proteins of GIT bacteria of multiple sclerosis patients with FDA approved drug benzodiazepine.

(a) [*Acinetobacter calcoaceticus*](https://www.uniprot.org/taxonomy/471) I, (b) [*Acinetobacter calcoaceticus*](https://www.uniprot.org/taxonomy/471) II, (c) [*Acinetobacter calcoaceticus*](https://www.uniprot.org/taxonomy/471) III, (d) [*Acinetobacter calcoaceticus*](https://www.uniprot.org/taxonomy/471) IV, (e) [*Acinetobacter calcoaceticus*](https://www.uniprot.org/taxonomy/471) V, (f) [*Acinetobacter calcoaceticus*](https://www.uniprot.org/taxonomy/471) VI, (g) [*Clostridium* sp](https://www.uniprot.org/taxonomy/59620). I, (h) [*Clostridium* sp](https://www.uniprot.org/taxonomy/59620). II, (i) *Salmonella typhimurium*
